# Activation of the hypoxia response protects mice from amyloid-β accumulation

**DOI:** 10.1007/s00018-022-04460-6

**Published:** 2022-07-19

**Authors:** Teemu Ollonen, Margareta Kurkela, Anna Laitakari, Samuli Sakko, Henna Koivisto, Johanna Myllyharju, Heikki Tanila, Raisa Serpi, Peppi Koivunen

**Affiliations:** 1 Biocenter Oulu, Faculty of Biochemistry and Molecular Medicine, Oulu Center for Cell-Matrix Research, University of Oulu, Aapistie 7C, P.O. Box 5400, 90014 Oulu, Finland; 2 A.I. Virtanen Institute for Molecular Sciences, University of Eastern Finland, Kuopio, Finland

**Keywords:** Alzheimer’s disease, HIF, Hypoxia, Inflammation, Metabolism, Vascularity

## Abstract

Alzheimer’s disease (AD) is the most common cause of dementia with limited treatment options affecting millions of people and the prevalence increasing with the aging population. The current knowledge on the role of the hypoxia/hypoxia-inducible factor (HIF) in the AD pathology is restricted and controversial. We hypothesized based on benefits of the genetic long-term inactivation of HIF prolyl 4-hydroxylase-2 (HIF-P4H-2) on metabolism, vasculature and inflammatory response that prolonged moderate activation of the hypoxia response could hinder AD pathology. We used an aging model to study potential spontaneous accumulation of amyloid-β (Aβ) in HIF-P4H-2-deficient mice and a transgenic APP/PSEN1 mouse model subjected to prolonged sustained environmental hypoxia (15% O_2_ for 6 weeks) at two different time points of the disease; at age of 4 and 10 months. In both settings, activation of the hypoxia response reduced brain protein aggregate levels and this associated with higher vascularity. In the senescent HIF-P4H-2-deficient mice metabolic reprogramming also contributed to less protein aggregates while in APP/PSEN1 mice lesser Aβ associated additionally with hypoxia-mediated favorable responses to neuroinflammation and amyloid precursor protein processing. In conclusion, continuous, non-full-scale activation of the HIF pathway appears to mediate protection against neurodegeneration via several mechanisms and should be studied as a treatment option for AD.

**Graphical abstract:**

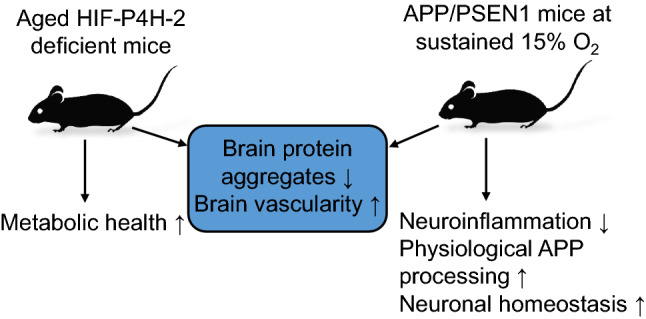

**Supplementary Information:**

The online version contains supplementary material available at 10.1007/s00018-022-04460-6.

## Introduction

Alzheimer’s disease (AD) is the most common neurodegenerative disease affecting millions of people worldwide characterized by age-associated progressive deterioration of neuronal structures and functions ultimately leading to cognitive disability and dementia [1, 2]. A central pathological feature of AD is the failure to clear insoluble amyloid-β (Aβ) peptide, predominantly manifesting in the cerebral cortex and hippocampus, followed by formation of neurofibrillary tangles. AD can be categorized to an early-onset (average ~ 45 years) genetically driven familial form accounting for less than 1% of the cases and to late-onset (over 65 years) sporadic form [1–3]. Whereas in the early-onset AD, the underlying cause can be pinpointed to increased Aβ production due to gene mutations in its precursor, amyloid precursor protein (*APP*), or APP-cleaving presenilin 1 or 2 (*PSEN1, PSEN2)* [1–3], the pathogenesis of the sporadic disease form appears to be a multifactorial cascade involving genetic, health and environmental factors, such as heart disease, obesity and associated diseases, inflammatory responses and reactive oxygen species [1–3]. According to current knowledge, the misfolded proteins in the aging brain result in oxidative and inflammatory damage, which in turn leads to energy failure and synaptic dysfunction.

Oxygen homeostasis is essential for normal physiology and many diseases are complicated by hypoxia. Transcriptional response of cells to hypoxia is chiefly mediated by the hypoxia-inducible factor (HIF)/HIF hydroxylase system, where three oxygen sensing HIF prolyl 4-hydroxylases (HIF-P4Hs, also known as PHDs/EGLNs) target the HIFα subunit (HIF1α-3α) to degradation via von Hippel Lindau ubiquitin ligase when O_2_ is available [4]. Under hypoxia the catalytic activity of HIF-P4Hs is inhibited resulting in a stabilized HIFαβ dimer which can initiate the transcription of hundreds of HIF target genes involved e.g., in erythropoiesis, angiogenesis, energy metabolism, inflammatory and immunological responses and regeneration [4]. In general, the physiological HIF response is a cellular rescue mechanism. HIF can also be stabilized under normoxia with small molecule HIF-P4H inhibitors which have been approved for the treatment of renal anemia [5, 6]. Preclinical data have, however, suggested that HIF-P4H inhibition could be beneficial in conditions beyond anemia, such as ischemia, metabolic dysfunction, fatty liver disease, atherosclerosis and inflammatory conditions many of which associate with AD [7, 8].

The current knowledge on the role of the hypoxia response/HIF in the AD pathology is limited and partly controversial. Acute and intermittent hypoxia have been associated with AD exacerbation involving e.g., increased β-site APP-cleaving enzyme 1 (BACE1) levels, altered APP processing, induced autophagy and increased Aβ accumulation [9–15]. HIF1 has been also suggested to compromise mitochondrial metabolism resulting in dysfunction of the microglia [16]. On the other hand, acute or chronic hypoxia have also been reported not to alter APP processing [17], and HIF1α has recently been associated with enhanced synaptosome and Aβ phagocytosis [18]. It is conceivable that the effects of hypoxia and activation of the HIF response may vary due to their magnitude and onset of the disease course, and it is also unclear whether they are a cause or a consequence of AD.

We hypothesized that systemic long-term activation of the HIF response, either via genetic inhibition of the most abundant isoenzyme HIF-P4H-2 or by prolonged moderate environmental hypoxia, could hinder AD pathology. We used an aging model to study the spontaneous accumulation of Aβ protein aggregates (mimicking the sporadic form of AD) in HIF-P4H-2-deficient mice and a transgenic mouse model overexpressing amyloidogenic human *APP* and *PSEN1* variants (APP/PSEN1) subjected to sustained environmental hypoxia (15% O_2_ for 6 weeks) at two age points, 4 and 10 months. Our data show that in both models, long-term activation of the hypoxia response resulted in less protein aggregate accumulation in the brain. Better brain vascularity was associated with less aggregates in both models. In the senescent HIF-P4H-2-deficient mice healthier metabolism associated with the protection, while in the hypoxia-treated APP/PSEN1 mice, the reduced neuroinflammation, lower BACE1 levels, enhanced physiological processing of APP and gene expression changes promoting neuronal homeostasis upon aging also contributed to less Aβ.

## Results

### Spontaneous age-associated protein aggregates in the brain of senescent mice are reduced by HIF-P4H-2 deficiency

**Fig. 1:**
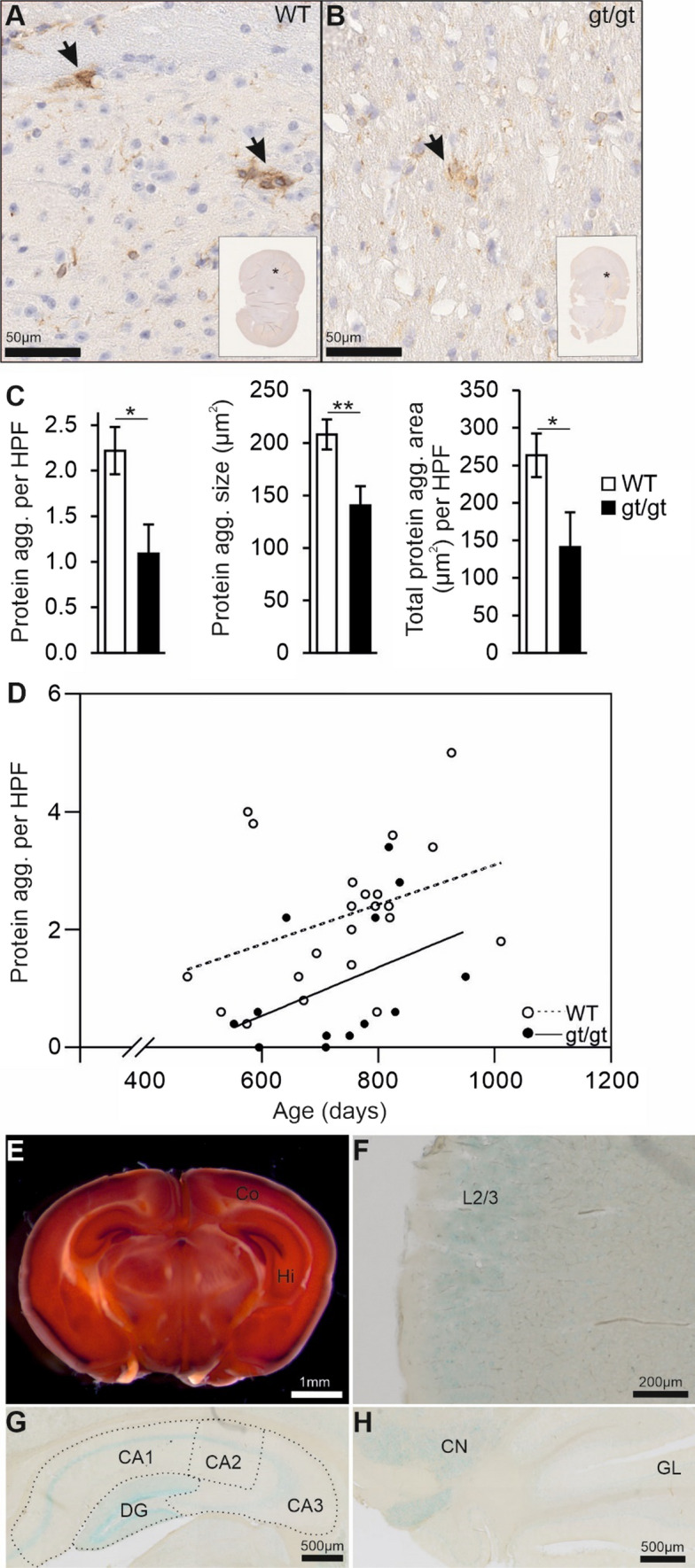
HIF-P4H-2 deficient mice display less protein aggregates in the brain in senescence. **A, B** Aged-matched Aβ-stained senescent wild-type (WT) and *Hif-p4h-2*^*gt/gt*^ (gt/gt) brain tissue. Scale bars = 50 µm. The brain regions of the enlarged pictures are shown in the inset by an asterisk. Arrows indicate the Aβ-positive protein aggregates. **C** Number of Aβ-positive protein aggregates, the average size of individual aggregates and the total aggregate-covered area per HPF. Five HPF/mouse were analyzed. **D** Correlation of the number of Aβ-positive protein aggregates with age. **E** X-gal-stained coronal gt/gt brain section. Scale bar = 1 mm. **F** X-gal-stained cross-section of the auditory cortex in a gt/gt mouse. The positivity is largely limited to layer 2/3. Scale bar = 200 µm. **G, H** X-gal-stained coronal gt/gt hippocampus and coronal cerebellum, respectively. Scale bars = 500 µm. Data are means ± SEM. *P < 0.05, **P < 0.01 in *T*-test. In **C** and **D**, *n* = 22 WT, 13 gt/gt males. *agg.* Aggregate, *CA* cornu ammonis, *CN* cerebellar nuclei, *Co* cortex, *DG* dentate gyrus, *GL* granular layer, *Hi* hippocampus, *HPF* high-powered field, *L2/3* layer 2/3

Histological inspection of a senescent male mice cohort, aged until they reached a humane endpoint at average of 2-years [19], revealed morphological alterations in brain resembling senile plaques typical for AD. Immunohistological staining indicated that these protein aggregates were positive for Aβ, their number increased with age starting from ~ 500 days but their number and area in the brain of the HIF-P4H-2 hypomorphic (*Hif-p4h-2*^*gt/gt*^) mice were significantly lower than in the wild type (wt) (Fig. 1A–D). To our knowledge, spontaneous accumulation of Aβ in aged wt mice has not been reported earlier and we therefore verified the specificity of the antibody (ab2539, anti-mouse/human Aβ) against Aβ with the widely used 6E10 Aβ recognizing antibody in the APP/PSEN1 mouse brain (Supplemental Fig. 1). However, we were unable to receive the same signal in the senescent brain with any of four other commercially available Aβ recognizing antibodies tested and therefore cannot be absolutely certain the protein aggregates were of Aβ. We have earlier reported that *Hif-p4h-2*^*gt/gt*^ mice have a HIF stabilization driven metabolic switch promoting glucose intake and glycolysis over oxidative phosphorylation and larger capillaries in the heart and skeletal muscle. Furthermore, these changes associate with improved quality of life in senescence compared to wt manifesting as lower body weight and adiposity and a lower incidence of liver diseases (including cancer), inflammation and spontaneous non-fatal myocardial infarctions [19–22].

As the knowledge on the role of HIF-P4H-2 in the brain or in AD pathogenesis is very limited, we first studied its expression in the brain exploiting the β–gal reporter under the endogenous *Hif-p4h-2* promoter in the *Hif-p4h-2*^*gt/gt*^ mice. X-gal staining showed HIF-P4H-2 to be expressed in the granule cell layer of the cortex, in the CA1-3 regions and dentate gyrus of the hippocampus and cerebellar nuclei (Fig. 1E–H); areas relevant for AD. We next studied the knockdown level of HIF-P4H-2 in the *Hif-p4h-2*^*gt/gt*^ brain. The level of wt *Hif-p4h-2* mRNA expressed in the *Hif-p4h-2*^*gt/gt*^ whole brain lysate was about 57% of wt and the levels in dissected cortex, hippocampus and cerebellum 60%, 65% and 64%, respectively (Supplemental Fig. 2A). Therefore, the knockdown of ~ 40% in the brain was less than those in most other tissues ranging from 65 to 90% [19] but sufficient to result in a faint normoxic stabilization of HIF1α in the *Hif-p4h-2*^*gt/gt*^ hippocampus (Supplemental Fig. 2B).

### HIF mediated alterations to metabolism and vasculature associate with protection against brain protein aggregate accumulation

**Fig. 2:**
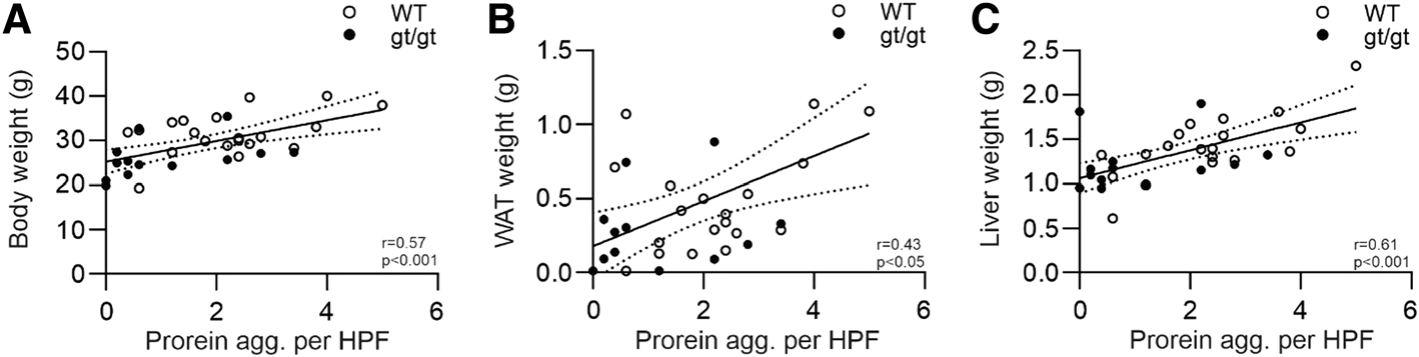
Number of age-associated brain protein aggregates correlates with the body weight and metabolic tissue weights of the senescent HIF-P4H-2 deficient mice. **A** Correlation of wild-type (WT) and *Hif-p4h-2*^*gt/gt*^ (gt/gt) body weight with the number of brain protein aggregates. **B**, **C** Correlation of the WAT and liver weight, respectively, with the number of brain protein aggregates. *n* = 22 WT, 12–13 gt/gt. *agg.* Aggregate, *HPF* high-powered field, *WAT* white adipose tissue

We next assessed whether the general metabolic parameters in the senescent wt and *Hif-p4h-2*^*gt/gt*^ mice correlate with the brain protein aggregates. Indeed, the extent of the brain protein aggregates correlated positively with body weight, white adipose tissue (WAT) weight and liver weight (Fig. 2A–C). We have earlier reported that these values in *Hif-p4h-2*^*gt/gt*^ mice are significantly from 25 to 60% lower than in wt mice [19]. These data suggest that the lower body weight, adiposity and liver weight in the senescent *Hif-p4h-2*^*gt/gt*^ mice may protect against neurodegeneration.

**Fig. 3:**
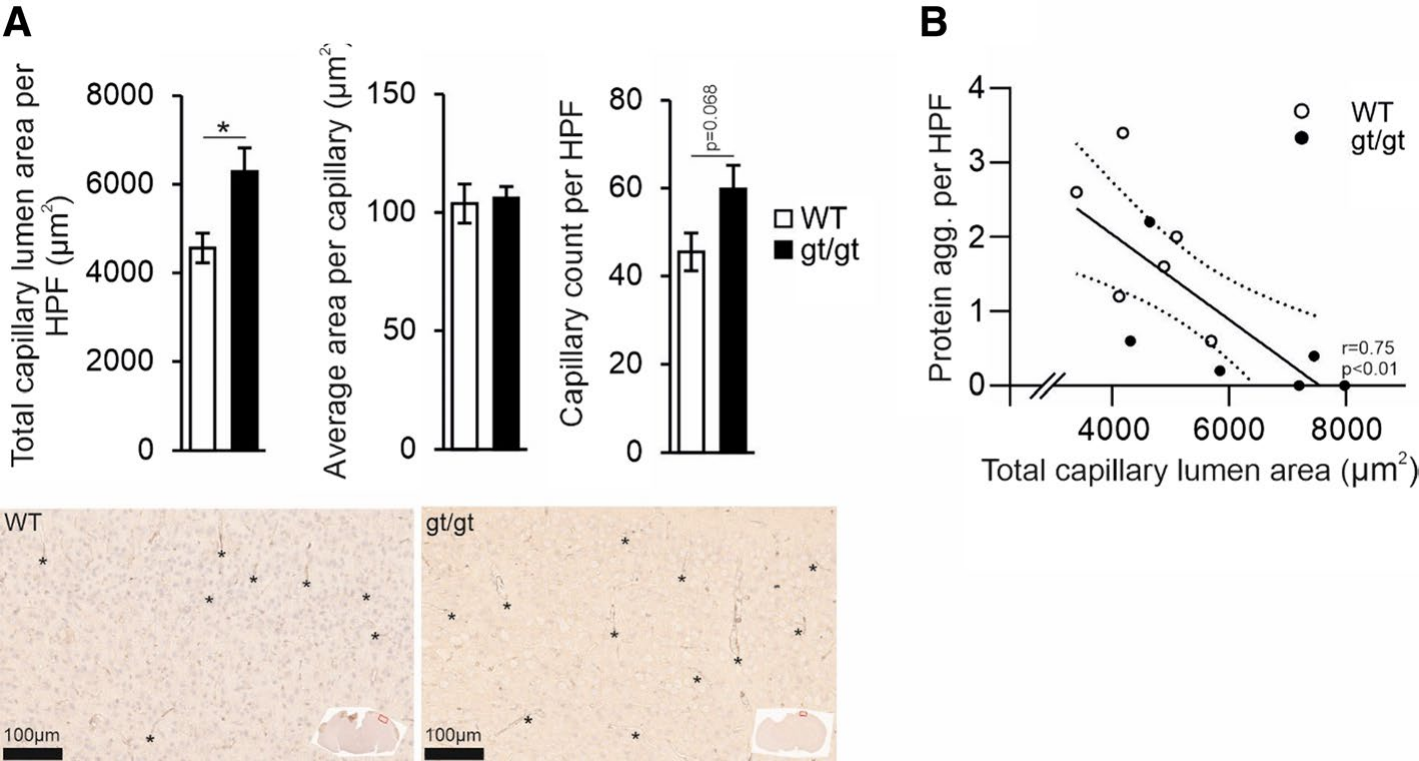
Increased capillary lumen area associates with lower number of age-associated brain protein aggregates in the HIF-P4H-2 deficient mice. **A** The total capillary lumen area, the average size of a capillary lumen and the number of individual capillaries analyzed in senescent age-matched wild-type (WT) and *Hif-p4h-2*^*gt/gt*^ (gt/gt) brain sections with GLUT1 staining. In the histological pictures the brain regions of the enlarged pictures are shown in the inset by a red square. Asterisks indicate examples of capillaries. **B** Correlation of the number of brain protein aggregates with the total capillary lumen area. Data are means ± SEM. *P < 0.05 in *T*-test. *n* = 6 WT, 6 gt/gt. *agg.* Aggregates, *HPF* high-powered field

Immunohistological analysis of GLUT1 expression, which colocalizes predominantly to the capillary endothelium in the brain, indicated that the senescent *Hif-p4h-2*^*gt/gt*^ mice have a larger total area of brain capillaries than wt mice, mainly due to a higher capillary count than a larger capillary area (Fig. 3A–C). There was a significant negative correlation between the protein aggregates and the capillary area (Fig. 3D), suggesting that the higher vascularity in the *Hif-p4h-2*^*gt/gt*^ brain associated with lesser neurodegeneration. Altogether, these data suggest that two key HIF mediated responses, induced angiogenesis and metabolic reprogramming, contributed to less age-associated brain protein aggregates in senescence.

### Prolonged sustained environmental hypoxia protects APP/PSEN1 mice from AD-associated neuroinflammation

**Fig. 4:**
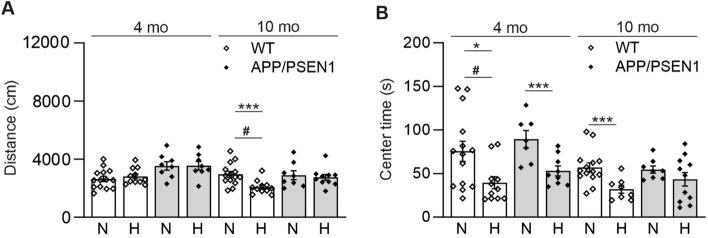
Hypoxia treatment decreases the time spent in the center of the open field. Behavioral assessment of wild-type (WT) and APP/PSEN1 mice after 4 weeks of hypoxia/normoxia treatment. **A** The distance traversed and **B** the time spent in the open field center. Data are means ± SEM. #*P* > 0.05 in 3-way ANOVA, *P < 0.05, ***P < 0.005 in *T*-test, *#P* < 0.05 in 3-way ANOVA. *n* = 14 WT N 4 mo, *n* = 11 WT H 4 mo, *n* = 7–8 APP/PSEN1 N 4 mo, *n* = 8–9 APP/PSEN1 H 4 mo, *n* = 14 WT N 10 mo, *n* = 9–11 WT H 10 mo, *n* = 8 APP/PSEN1 N 10 mo, *n* = 10–11 APP/PSEN1 H 10 mo. *H* hypoxia, *mo* months old, *N* normoxia

We next studied whether amyloid plaque producing APP/PSEN1 mice would show protection against Aβ accumulation and neurodegeneration when exposed to continuous normobaric 15% O_2_ for 6 weeks instead of normal 21% O_2_. We studied the effect of such intervention in two age cohorts of APP/PSEN1 and wt male mice; 4-month-old (appearance of the first amyloid plaques) and 10-month-old (fully developed amyloid pathology). Four weeks after the intervention start the mice underwent a 10-min open field test. The hypoxic intervention reduced the distance 10-month-old wt males traversed compared to normoxia (Fig. 4A). In addition, among both genotypes, the hypoxia exposed mice spent less time in the arena center compared with normoxia, seen more clearly in the younger cohort (Fig. 4B). These data suggest that hypoxia exposure made the mice more scared and careful, as mice normally prefer to avoid open places.

**Fig. 5:**
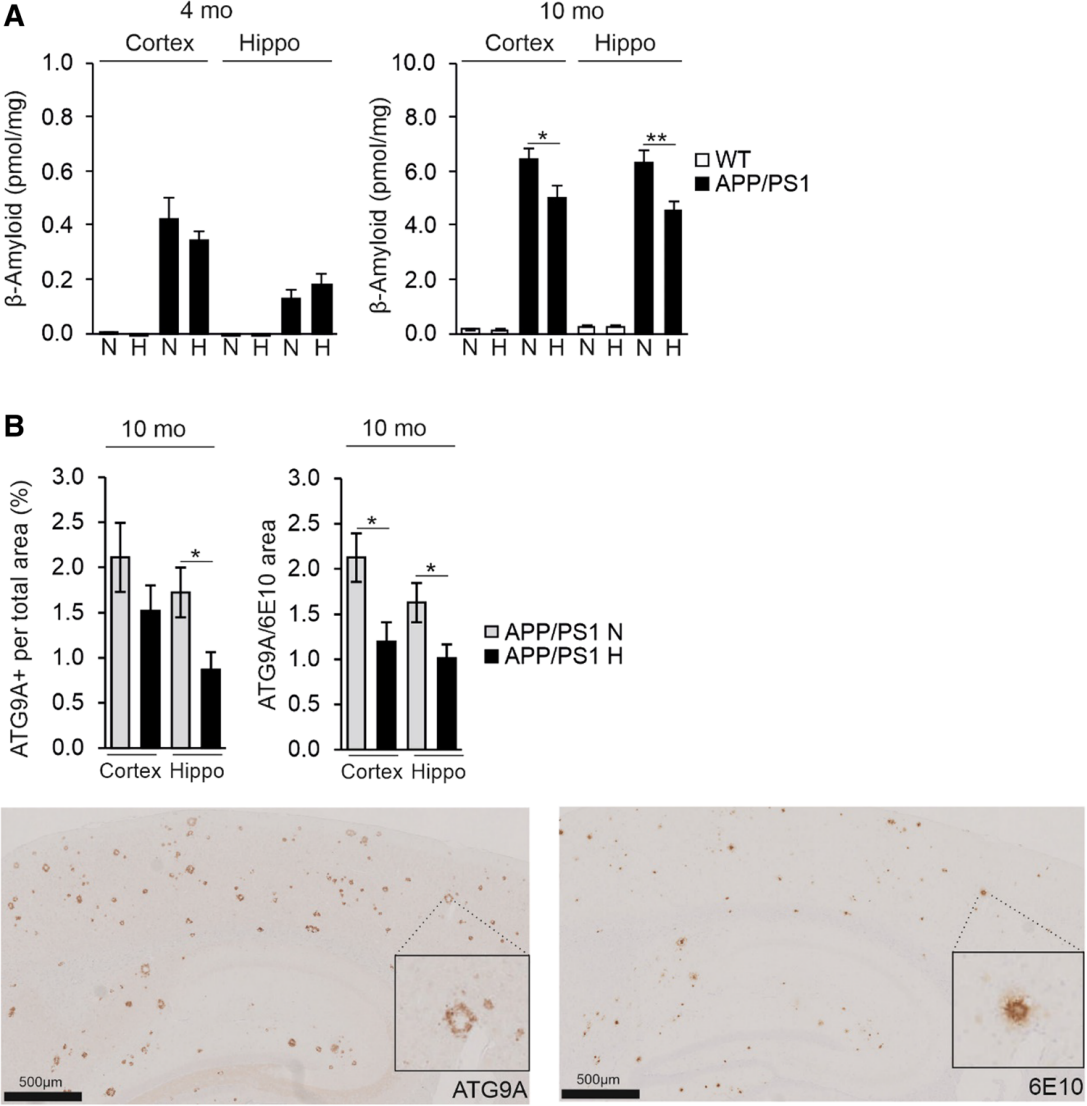
Hypoxia treatment decreases brain Aβ levels and the area of dystrophic neurites colocalizing with the Aβ plaques in the APP/PSEN1 mice. **A** Aβ amount in the cortex and hippocampus of 4 and 10 mo WT and APP/PSEN1 mice kept for 6 weeks in normoxia or hypoxia. **B** ATG9A-positive total area and ATG9A-positive area colocalizing with Aβ–positive area detected by 6E10 antibody in the brain of 10-month-old APP/PSEN1 mice under normoxia and hypoxia. Example pictures of histological stainings of consecutive sections of ATG9A and 6E10 in cortex and hippocampus, respectively. Data are means ± SEM. *P < 0.05, **P < 0.01 in *T*-test. *n* = 14 WT N 4 mo, *n* = 12 WT H 4 mo, *n* = 8 APP/PSEN1 N 4 mo, *n* = 9–10 APP/PSEN1 H 4 mo, *n* = 14 WT N 10 mo, *n* = 11 WT H 10 mo, *n* = 8–9 APP/PSEN1 N 10 mo, *n* = 9–11 APP/PSEN1 H 10 mo. *Hippo* hippocampus, *H* hypoxia, *mo* months old, *N* normoxia

At sacrifice, the amount of Aβ measured in the homogenates of the cortex and hippocampus was low in the 4-month-old APP/PSEN1 mice, with no difference between the normoxia and hypoxia groups (Fig. 5A). The amount of Aβ was markedly higher in the 10-month-old APP/PSEN1 cortex and hippocampus compared to 4 months, however, the levels in the hypoxia group were significantly, ~ 20%, lower than in normoxia (Fig. 5A). Of note, the level of Aβ in the brain of the wt controls was not detectable at 4 months and just above the detection limit at 10 months of age (Fig. 5A). In immunohistological analyses, there was no significant difference in plaque size between the groups (Supplemental Fig. 3A, B). However, a trend for a higher cellular density in the Aβ-deposit-free area was seen in the hypoxic APP/PSEN1 cortex and hippocampus compared to normoxia in both age groups while this parameter declined in all groups with aging (Supplemental Fig. 3C). No significant difference was detected in the cellular density in Aβ-deposit-affected area between any groups (Supplemental Fig. 3D). We then detected with ATG9A immunohistochemical staining the area of dystrophic neurites, a specific and sensitive indicator of neuronal damage, and its colocalization with the Aβ deposits. The total ATG9A-positive area was 50% less in the hippocampus and the ATG9A-positive colocalizing with the Aβ deposits ~ 40% less in the hypoxia treated cortex and hippocampus of the 10-month-old APP/PSEN1 mice compared with normoxia (Fig. 5B). Thus, the hypoxic exposure had reduced neurodegeneration.

**Fig. 6:**
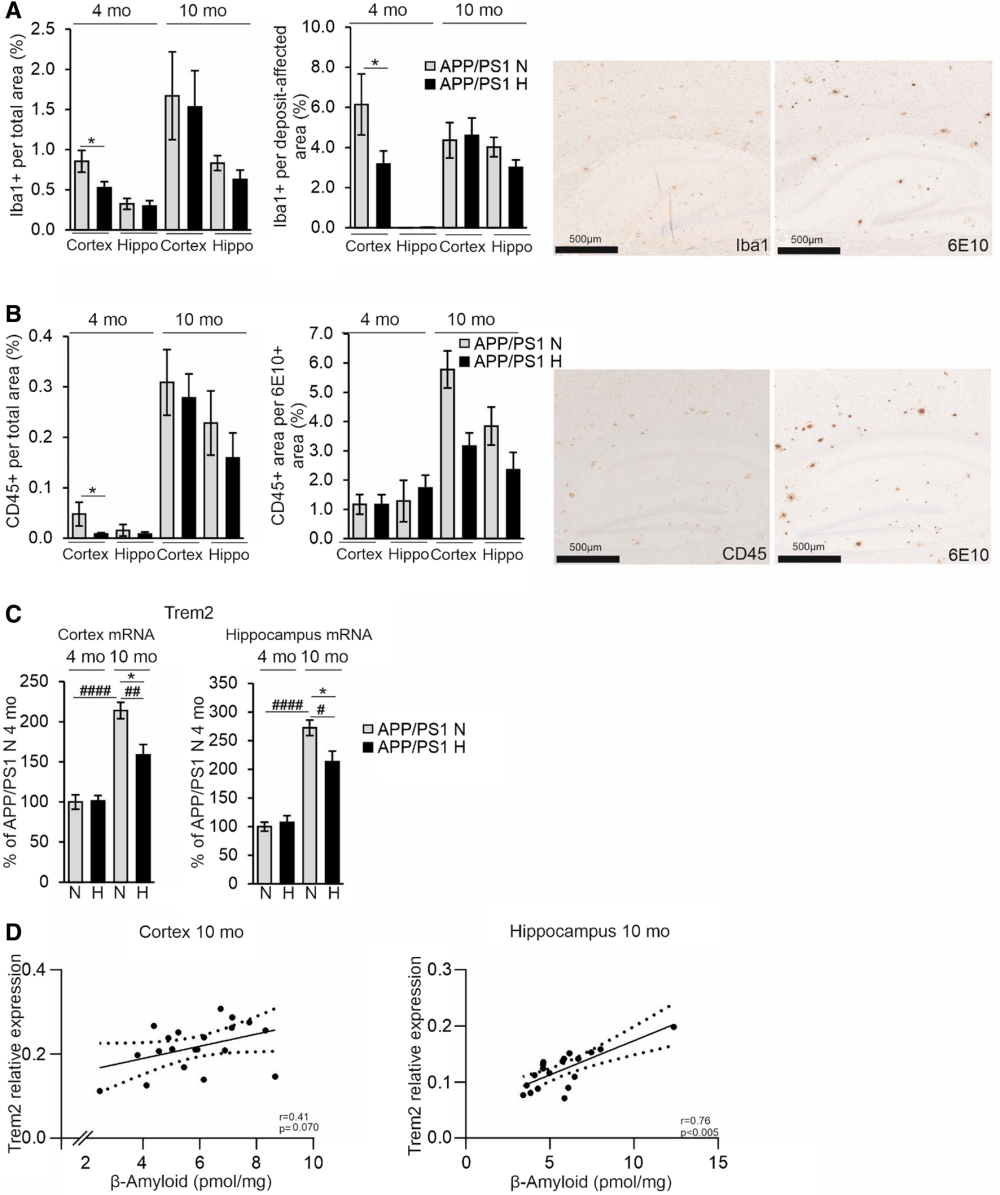
Hypoxia treatment decreases brain neuroinflammatory responses in the APP/PSEN1 mice. **A** IBA1-positive area total area and IBA1-positive area in relation to the Aβ deposit-affected area. Aβ-positive deposits were detected by 6E10 antibody. Example pictures of histological stainings of consecutive sections of IBA1 and 6e10 in cortex and hippocampus, respectively. **B** CD45-positive total area and CD45-positive area in relation to Aβ area detected by 6E10 antibody. Example pictures of histological stainings of consecutive sections of CD45 and 6E10 in cortex and hippocampus, respectively. **C** qPCR analysis of the mRNA levels of *Trem2* in the cortex and the hippocampus of 4 and 10 mo APP/PSEN1 mice. **D** Correlation of the *Trem2* mRNA relative expression with the Aβ amount in the cortex and hippocampus of the 10 mo APP/PSEN1 mice. Data are means ± SEM. *P < 0.05 in *T*-test, *##P* < 0.01, ###*#P* < 0.0001 in 2-way ANOVA. *n* = 14 WT N 4 mo, *n* = 12 WT H 4 mo, *n* = 8 APP/PSEN1 N 4 mo, *n* = 9–10 APP/PSEN1 H 4 mo, *n* = 14 WT N 10 mo, *n* = 11 WT H 10 mo, *n* = 8–9 APP/PSEN1 N 10 mo, *n* = 9–11 APP/PSEN1 H 10 mo. *Hippo* hippocampus, *H* hypoxia, *mo* months old, *N* normoxia

Analysis of microglia with IBA1 in the cortex showed that the total IBA1-positive area and the IBA1-positive area in the vicinity of the amyloid plaques were significantly smaller in the hypoxia treated 4-month-old APP/PSEN1 mice compared to normoxia (Fig. 6A). No differences between the hypoxia and normoxia groups were detected in colocalization of IBA1 and ATG9A the cortex or hippocampus (Supplemental Fig. 3E). Since IBA1 does not distinguish between activated and homeostatic microglia, we carried out a further analysis by detecting CD45 which only stains the former. These analyses indicated that the total CD45-positive area was lower in all hypoxia treated brains compared to normoxia but this difference reached statistical significance only in the 4-month-old cortex (Fig. 6B). When assessed in relation to Aβ affected area, hypoxia treatment resulted in less CD45 positivity compared with normoxia in the 10-month-old brain but these differences were not significant (Fig. 6B). We also studied the levels of the microglial triggering receptor expressed on myeloid cells 2 (*Trem2)* mRNA and found it to be significantly upregulated in normoxia in APP/PSEN1 cortex and hippocampus at 10 months compared to 4 months while hypoxia significantly lowered its levels at 10 months (Fig. 6C). The extent of *Trem2* expression showed a significant positive correlation with Aβ levels in the hippocampus and a similar trend in the cortex (Fig. 6D). Altogether, these data showed that the hypoxia treatment of the APP/PSEN1 mice modulated microglial responses to protect against Aβ deposition and reduced the amount of dystrophic neurites in the plaques.

### Hypoxia-mediated vascular and lesser extent metabolic modifications convey protection from Aβ accumulation in APP/PSEN1 mice

Extensive analysis of the key metabolic parameters after the 6-week intervention showed trends (some reaching significance) for lower body weight, less adipocity, lower liver weight and reduced serum total cholesterol and HDL cholesterol levels in the hypoxia treated APP/PSEN1 and wt mice at both age groups compared to normoxia (Supplemental Fig. 4). The lower liver weight in hypoxia appeared to result mainly from the difference in liver glycogen levels in the younger mice while in the older mice also the lesser liver triglyceride content likely contributed to it (Supplemental Fig. 4). The APP/PSEN1 mice appeared resistant to the hypoxia-mediated trend for lower insulin levels and lesser insulin resistance seen in wt (Supplemental Fig. 4). The trend for higher serum triglycerides in the hypoxia treated mice independent of genotype associated with reduced WAT and liver weights, i.e., redistribution of fat (Supplemental Fig. 4). As a proof of concept, the hypoxia treatment significantly increased the blood hemoglobin (Hb) levels in all mice (Supplemental Fig. 4). Association analysis of the amount of brain Aβ and the metabolic parameters showed surprisingly few significant hits (Supplemental Table 1). Of these, the most significant was a positive correlation between cortical Aβ and liver glycogen (*r* = 0.55, *P* = 0.013) in the 10-month-old mice. An almost significant positive correlation was also observed between cortical Aβ and serum total cholesterol (*r* = 0.43, *P* = 0.068) in the 10-month-old mice. As a conclusion, the improved metabolic health of the hypoxia treated APP/PSEN1 mice did not markedly correlate with the reduced amount of brain Aβ.

**Fig. 7:**
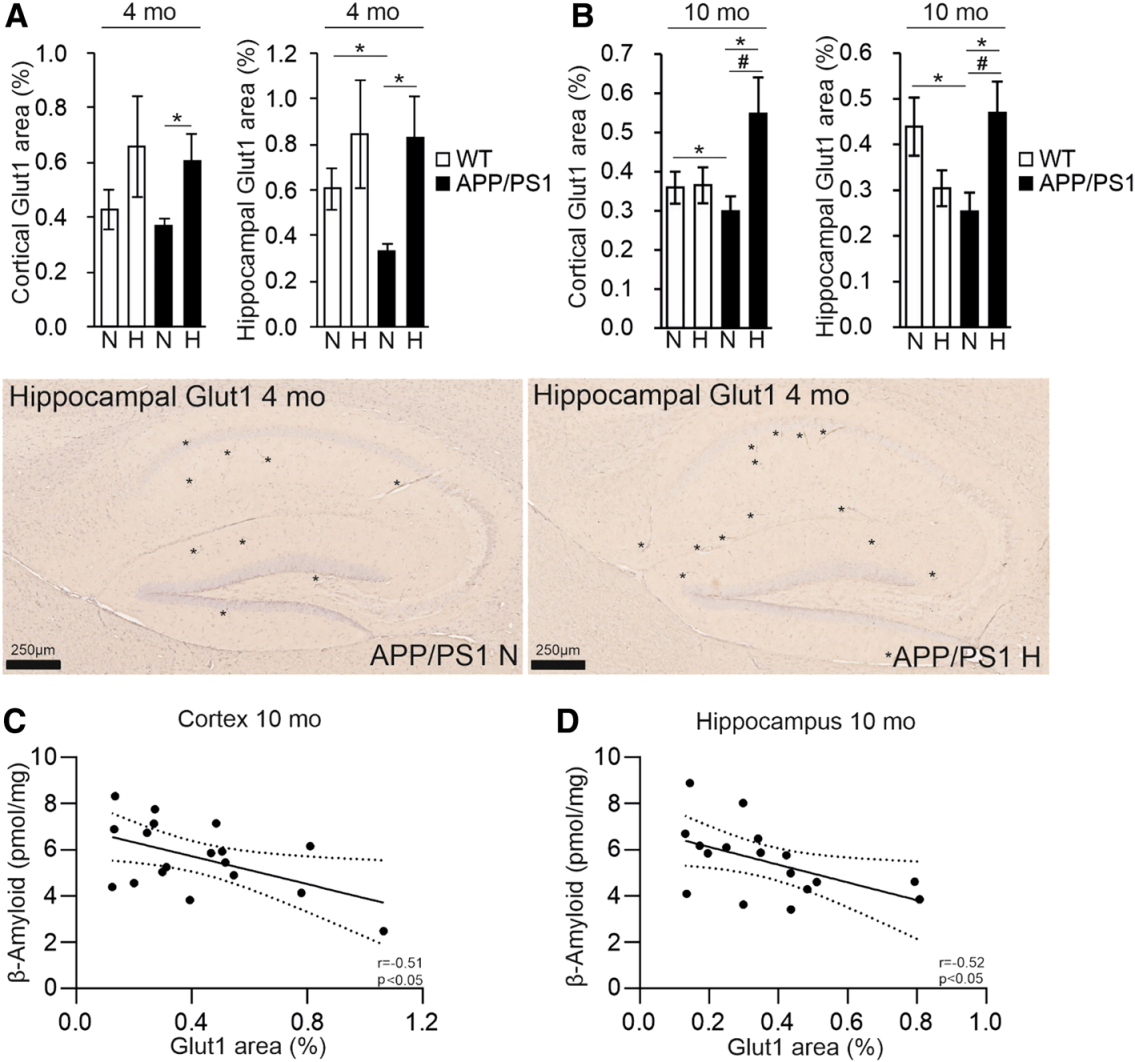
Hypoxia treatment increases the GLUT1-positive area in APP/PSEN1 brain and this associates with decreased Aβ amount. GLUT1-positive area per total cortical and hippocampal area of **A** 4 mo and **B** 10 mo mice treated for 6 weeks in normoxia or hypoxia. In the histological pictures asterisks indicate examples of Glut1-positive capillaries in the hippocampus. Correlation of Aβ amount with the GLUT1-positive area in **C** cortex and **D** hippocampus of 10 mo APP/PSEN1 mice. Data are means ± SEM. *P < 0.05 in *T*-test, *#P* < 0.05 in 2-way ANOVA. *n* = 5–6 WT N 4 mo, *n* = 5–6 WT H 4 mo, *n* = 5 APP/PSEN1 N 4 mo, *n* = 5–6 APP/PSEN1 H 4 mo, *n* = 11–14 WT N 10 mo, *n* = 11 WT H 10 mo, *n* = 9 APP/PSEN1 N 10 mo, *n* = 10–11 APP/PSEN1 H 10 mo. *Aβ* β-amyloid, *H* hypoxia, *mo* months old, *N* normoxia

In pairwise comparisons, there was a genotype-driven reduction in the GLUT1-positive vascular area in the APP/PSEN1 mice in the hippocampus at 4 and 10 months and in cortex at 10 months in normoxia (Fig. 7A, B). Although, increases in the vascular area were detected in both genotypes in hypoxia in the 4-month-old cortex and hippocampus, they only reached statistical significance in the APP/PSEN1 mice in pairwise comparisons to normoxia (Fig. 7A). In the 10-month-old mice hypoxia treatment increased the GLUT1-positive area in the APP/PSEN1 cortex and hippocampus but not in the wt brain (Fig. 7B). There were significant negative correlations between cortical and hippocampal Aβ and GLUT1-positive area in the 10-month-old cohort (Fig. 7C, D) suggesting that increased vascularity was protective against Aβ accumulation. Significant increase in the *Glut1* mRNA level by hypoxia was detected in the APP/PSEN1 cortex at 4 months while no significant differences in the *Vegfc* or *Vegfd* mRNA levels were observed at either age (Supplemental Fig. 5).

### Hypoxia increases APP processing toward the physiological pathway and supports maintenance of neuronal homeostasis upon aging via gene expression changes

**Fig. 8:**
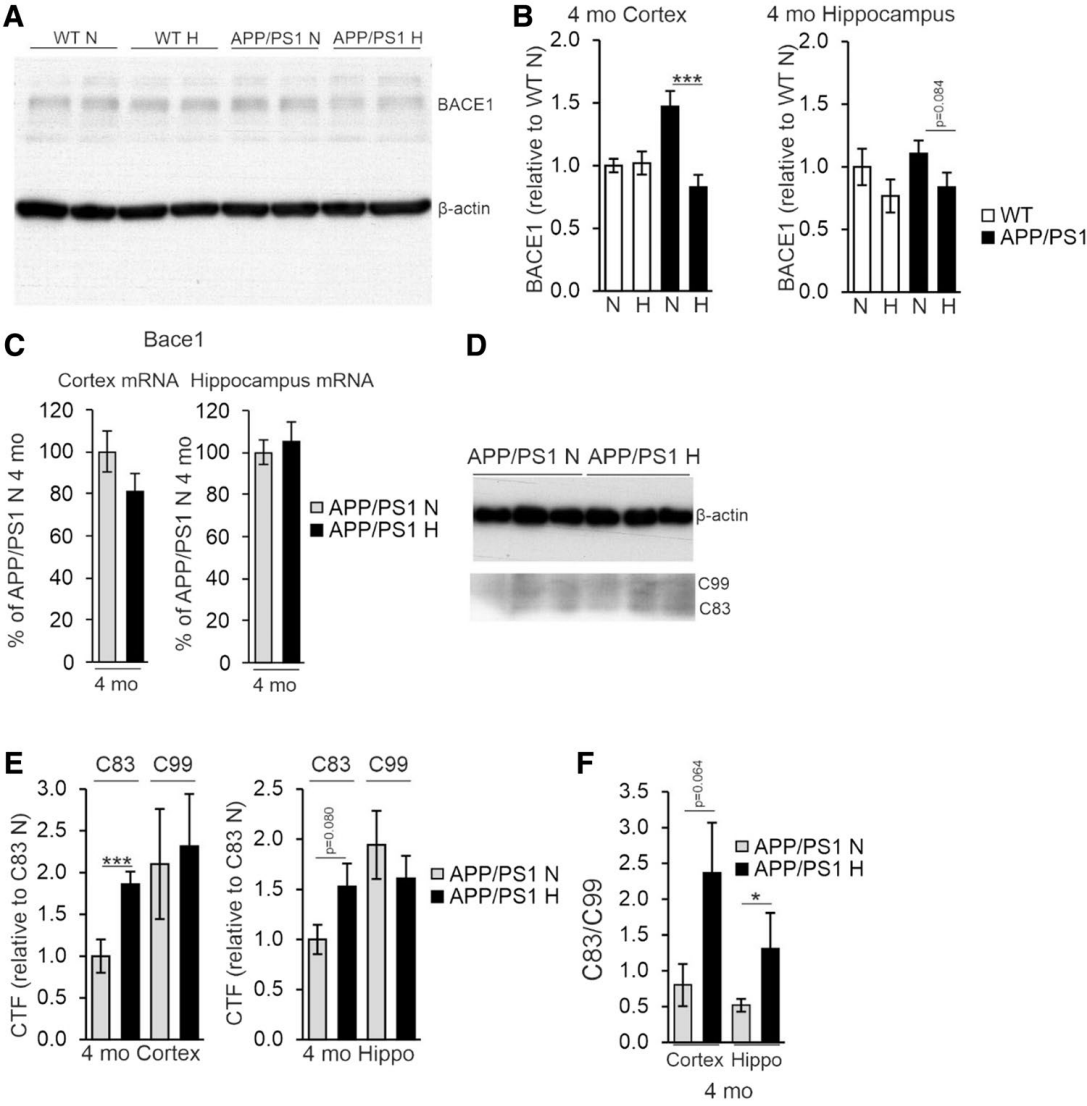
Hypoxia treatment alters the post-translational modification of APP. **A** Western blot analysis of BACE1 levels from 4 mo wild-type (WT) and APP/PSEN1 mice treated in normoxia or hypoxia for 6 weeks. **B** Quantification of the relative BACE1 relative protein levels in cortex and hippocampus of 4 mo APP/PSEN1 mice. **C** qPCR analysis of the *Bace1* mRNA levels in cortex and hippocampus of 4 mo APP/PSEN1 mice. **D** Western blot analysis of the levels of C-terminal factors (CTFs) of APP from 4 mo APP/PSEN1 mice. **E** Quantification of the relative CTF levels in cortex and hippocampus of 4 mo mice. **F** Ratio of APP CTFs in cortex and hippocampus of 4 mo APP/PSEN1 mice. Data are means ± SEM. *P < 0.05, ***P < 0.005 in *T*-test. *n* = 8 APP/PSEN1 N 4 mo, *n* = 9–10 APP/PSEN1 H 4 mo. *CTF* C-terminal factor, *hippo* hippocampus, *H* hypoxia, *mo* months old, *N* normoxia

To evaluate whether the detected lowered Aβ amount in the cortex and hippocampus of the hypoxia treated APP/PSEN1 mice involved altered processing of APP, we determined the level of BACE1 by Western blotting and found a significant > 40% decrease in BACE1 levels in the cortex of the hypoxia treated 4-month-old mice compared to normoxia and a similar trend in the hippocampus (Fig. 8A, B). No significant differences in *Bace1* mRNA levels were detected between the treatment groups (Fig. 8C), speaking for a post-translational effect of hypoxia. In contrast, no difference between the treatment groups was detected in the BACE1 protein or mRNA levels in the 10-month-old mice (data not shown). To further evaluate the hypoxia impact on APP cleavage, we determined the amounts of the non-amyloidogenic α-cleaved C83 and amyloidogenic β-cleaved C99 fragments by Western blotting at 4 months (Fig. 8D). We found a higher relative amount of C83 in the hypoxia treated APP/PSEN1 cortex and hippocampus compared with normoxia while no difference in the relative amount of C99 was detected resulting in an increased C83/C99 ratio (Fig. 8E, F).

**Fig. 9:**
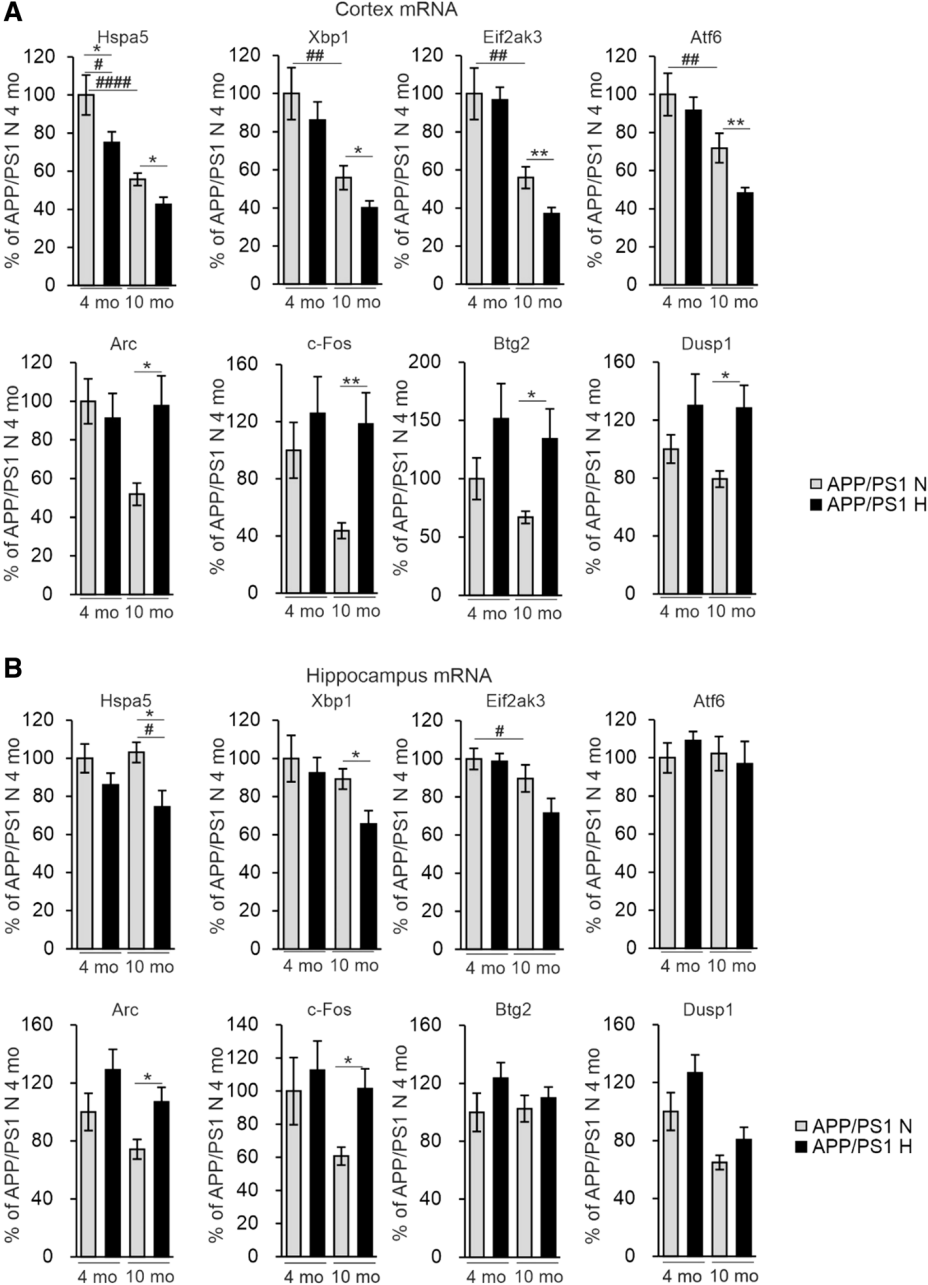
Hypoxia treatment modified gene expression to promote brain cell functions and survival. qPCR analysis of the mRNA levels of the indicated genes in **A** cortex and **B** hippocampus of the APP/PSEN1 mice treated for 6 weeks in normoxia or hypoxia. Data are means ± SEM. *P < 0.05, **P < 0.01 in *T*-test *#P* < 0.05, *#P* < 0.05, *##P* < 0.01, *####P* < 0.0001 in 2-way ANOVA. *n* = 8 N 4 mo, *n* = 9–10 H 4 mo, *n* = 8–9 N 10 mo, *n* = 10–11 H 10 mo

Hypoxia induces endoplasmic reticulum (ER) stress which has also been indicated as a trigger for inflammation and AD pathology [23, 24]. We therefore determined the mRNA levels of several ER markers in the APP/PSEN1 cortex and hippocampus and found significant hypoxia-associated reductions especially in pairwise comparisons in *Hspa5*, *Xbp1*, *Eif2ak3* and *Atf6* in the older cohort, and age-associated reductions in the same mRNAs in cortex in normoxia (Fig. 9A, B); however, none of these correlated with the amount of Aβ (Supplemental Table 1). To identify potential additional unprecedented mechanisms involved in the hypoxia-mediated alleviation of AD-like neuropathology we carried out RNA sequencing (RNASeq) analysis of the cerebral cortex of the 4-month-old hypoxia and normoxia treated APP/PSEN1 mice (*n* = 2 + 2). This analysis revealed a few differently expressed RNAs between the intervention groups among which we selected *Arc*, *c-Fos*, *Dusp1* and *Btg2* for further analyses by quantitative PCR (qPCR) in cortex and hippocampus (Supplemental Table 2). Interestingly, there were no differences in the mRNA levels of the neuronal plasticity associated *Arc* and the neuronal activity associated *c-Fos* mRNA between the normoxia and hypoxia groups at 4 months of age whereas in pairwise comparisons their levels in the APP/PSEN1 cerebral cortex and hippocampus at 10 months were significantly higher in hypoxia and comparable to the levels detected at 4 months (Fig. 9A, B). In pairwise analysis, hypoxia upregulated the anti-apoptotic *Dusp1* mRNA and the anti-inflammatory *Btg2* mRNA in 10-month APP/PSEN1 cortex but not hippocampus (Fig. 9A, B). None of these cortical mRNA levels correlated with the Aβ amount in the 10-month-old mice, but in hippocampus, there was a trend for negative correlations between Aβ and *Arc* and *c-Fos* mRNA, respectively (Supplemental Table 1), suggesting that their higher levels in hypoxia could mediate neuroprotection (Fig. 9B). Significant positive correlations were detected for cortical *c-Fos* (*r* = 0.48, *P* = 0.031) and *Btg2* (*r* = 0.51, *P* = 0.044) mRNA levels and cortical GLUT1, indicative for vascular density, respectively, in the 10-month-old mice (Supplemental Table 1). Altogether, these data suggest that the hypoxia treatment could alter gene expression especially in the aged APP/PSEN1 mice and contribute to maintenance of neuronal homeostasis especially upon aging.

## Discussion

The current literature gathered mostly from acute or oscillating hypoxia have mainly reported hypoxia/HIF to exacerbate AD pathology with a few exceptions. We hypothesized based on the benefits of the long-term inactivation of HIF-P4H-2 on metabolism, vascular function and inflammatory responses that sustained, moderate level activation of the HIF response could indeed alleviate AD pathology [19]. There is also epidemiological data which associate high altitude residency with lower mortality to metabolic and cardiac diseases predisposing to AD and to lower AD mortality [25, 26].

Sustained activation of the hypoxia response in two mouse models, genetic inhibition of HIF-P4H-2 and prolonged moderate environmental hypoxia in amyloid plaque forming APP/PSEN1 mice, indeed increased brain capillary area and reduced brain protein aggregate and Aβ levels, respectively. Moreover, in both models, the brain protein aggregate levels showed a negative correlation with observed higher vascularity, suggesting that these two changes are directly related to each other. The higher vascularity in the *Hif-p4h-2*^*gt/gt*^ brain agrees with our earlier findings in the heart and skeletal muscle where the larger capillaries and better perfusion in these mice protects against acute ischemia [22, 27]. GLUT1, responsible for transfer of glucose across the blood brain barrier, is selectively expressed at high levels in the brain capillary endothelium and has therefore been used as a marker for vascularization. The amount of GLUT1 is decreased in the hippocampus and cerebral cortex of AD patients [28]. Moreover, *GLUT1* is also a central HIF1 target gene supporting the glycolytic switch of metabolism occurring under hypoxia. Therefore, the here detected larger GLUT1-positive area, indicative of larger vascularity, may also have contributed to protection from hypometabolia. We also found a hypoxia-induced increase in the mRNA levels of immediate early genes *Arc* and *c-Fos,* the latter correlating with higher vascularity, and the anti-apoptotic gene *Dusp1*. These data suggest that signaling routes promoting neuronal cell activity, plasticity and survival were also upregulated by sustained, moderate hypoxia, and likely involved in the mediation of the neuroprotective effects of the HIF pathway.

In the APP/PSEN1 mouse line, the hippocampal vascularization was reduced at the age of 4 months compared to wt, agreeing with earlier data [29], and suggesting that hypoperfusion may also contribute to amyloid pathology in familial AD. Interestingly, the 6-week hypoxia treatment was able to increase the vascularization above the level of normoxic wt mice even when started at the age of 10 months, suggesting that the hypoxia pathway may be a modifiable factor regarding the vascular system even at later stages of the disease process.

BACE1 is the key rate-limiting enzyme for the production of Aβ [1, 2]. A hypoxia response element has been characterized in the regulatory region of *BACE1* and in cellular experiments acute hypoxia has been shown to upregulate its expression mainly HIF1-dependently [9–11]. However, earlier reports in the APP/PSEN1 mice using a very short (4 or 16 h) or prolonged (21 days) harsh hypoxic exposure (9% O_2_) found no substantial impact of hypoxia on Aβ generation [17]. However, similarly to our results here, a significant 40% reduction in brain BACE1 protein levels was seen after 16 h of the hypoxic exposure while the reduction of 30% after 21 days did not reach significance [17]. No difference in the *Bace1* mRNA levels was reported after hypoxia [17], agreeing with our data, and suggesting that the regulation of BACE1 levels in the APP/PSEN1 mice by hypoxia occurs at post-translational level likely involving protein stability. Despite reduced BACE1 levels, no difference in the total amount of the amyloidogenic C99 fragment of APP was detected here but instead an increase in the physiological C83 fragment. This likely stems from more active α-secretase in the hypoxic brain. It is also worth noticing that the decrease in BACE1 protein levels by hypoxia was most pronounced in the cortex of 4-month-old APP/PSEN1 mice that did not show a significant reduction in amyloid plaque load, while BACE1 was not decreased in 10-month-old mice with the most robust reduction of brain amyloid plaque load. Therefore, the changes in BACE1 levels were unlikely the main underlying mechanism for reduced brain amyloid load.

Microglial cells are specialized macrophage-like cells of the central nervous system involved in neuroinflammatory responses but also having functions in brain protection and repair including phagocytotic actions [30]. Microglia responds to Aβ accumulation by acquiring a unique transcriptional and functional signature called disease-associated microglia (DAM) [30]. DAM attenuate the progression of neurodegeneration in certain mouse models, but inappropriate DAM activation accelerates neurodegenerative disease in other models. TREM2, which is highly specific to microglia, is essential for the conversion of microglial toward the DAM profile and to respond to Aβ-plaque-induced pathology. Certain *TREM2* gene variants have been shown to substantially increase the risk of developing late-onset AD [30]. TREM2 has a dual role in brain amyloid deposition. TREM2 knockout is associated with increased amyloid seeding [31], while antibodies activating the TREM2 signaling reduce amyloidogenesis [32]. On the other hand, the most common TREM2 R47H variant does not change brain amyloid load but disrupts the microglia barrier around plaques and leads to more severe damage to surrounding axon terminals [32]. Similarly, HIF1 was recently reported to compromise mitochondrial metabolism resulting in dysfunction of the microglia [16]. On the other hand, HIF1α was associated with enhanced synaptosome and Aβ phagocytosis [18]. Following the chronic hypoxia treatment, we detected fewer microglia in the vicinity of the Aβ plaques and fewer total microglia in the cortex of the younger APP/PSEN1 cohort compared to normoxia but this difference was not seen in the older 10-month-old mice. On the other hand, we observed that *Trem2* expression was dampened in the 10-month-old APP/PSEN1 mice due to hypoxia, but hypoxia had no effect in the 4-month-old APP/PSEN1 mice. Therefore, it is unlikely that alternations in *Trem2* expression were driving the changes in microglia in the hypoxia group. Rather, the decreased *Trem2* expression in the 10-month-old hypoxia group simply reflects the hypoxia-induced decrease in its trigger, the amyloid plaque load. This contention is supported by the significant positive correlation between *Trem2* expression and brain Aβ levels both in the cortex and hippocampus. We also identified through the unbiased RNASeq screen and verified by qPCR analysis the anti-inflammatory *Btg2* as a novel hypoxia regulated mRNA in the APP/PSEN1 brain associated with higher vascular density which associates with less Aβ. Altogether data from us and others suggest activation of the hypoxia/HIF response affects brain microglia; the effects depend on the magnitude and the onset of the hypoxic response and may mediate protective or exacerbating effects.

Finally, it should be emphasized that despite of the partially identical outcome, the hypoxia response-associated study settings used here are not comparable to each other. In the *Hif-p4h-2*^*gt/gt*^ mice, the HIF response was activated without oxygen deprivation. This model offers a setting to study specifically the contribution of chronic life-long broad spectrum HIF activation to neurodegeneration. Since the knockdown level of HIF-P4H-2 in the *Hif-p4h-2*^*gt/gt*^ brain was lower than that in peripheral tissues, it is likely that the reprogramming of the peripheral metabolism to favor glycolysis [19] also contributed to the observed protection against age-associated protein accumulation in the brain. This was further supported by the negative associations of body weight and WAT and liver weights, respectively, which we have shown to account from differences in peripheral metabolism [20], with the brain protein aggregate levels. These data suggest that especially in sporadic AD healthier metabolism, mediated for example by activation of the HIF response, could offer protection from Aβ deposits. The 15% O_2_ concentration used here in the familial AD model equals altitude of 2700 m with permanent residents in several continents. It can be considered moderate hypoxia, and is significantly less hypoxic than e.g., the 8%, 9% O_2_ or 11% used in several studies [15–17] equaling 7700, 6700 or 5000 m of altitude, respectively, which would require oxygen supplementation for humans to survive. The global hypoxia-induced simultaneous inactivation of all HIF-P4Hs in comparison to HIF-P4H-2 alone may also result in a too full-burst HIF activation and counter effects, as seen e.g., in pVHL knockouts [33]. The environmental hypoxia do not only stabilize HIFα but it also inhibits other oxygen-sensitive enzymes/processes, such as chromatin modifications by histone demethylases [34, 35]. Although, the APP/PSEN1 mice represent the rare familial form of AD the findings from this model, as seen here, were partly overlapping with the aging-associated sporadic disease. Yet, it offers a preclinical setting for studying a common human disease and identification of potential new therapeutic targets, as confirmed here.

## Materials and methods

### Animal experiments

All the experiments were performed according to protocols approved by the Animal Experiment Board of Finland (ESAVI-8179-04.10.07-2017). *Hif-p4h-2*^*gt/gt*^ mice were generated with a GeneTrap targeting vector introduced into intron 1 of the *Hif-p4h-2* gene as previously described [36]. The wt littermates for the *Hif-p4h-2*^*gt/gt*^ mice were obtained from heterozygous matings. Male *Hif-p4h-2*^*gt/gt*^ mice were allowed to age until they reached humane endpoint or exactly 1-year. The mice were sacrificed, blood was collected from *vena cava* and the organs were collected. Age-matched groups were used in the analyses.

APPswe/PS1dE9 (APP/PSEN1) tg/wt male mice and their wt littermates of 4 and 10 months of age were subjected for six weeks to 15% O_2_ in a hypoxia workstation (Hypoxic Glove Box, Coy Laboratory Products, USA) (normobaric environmental hypoxia) or 21% O_2_ (normoxia). After four weeks of the treatment, behavioral assessment was carried out using an open field test [37]. Mice were placed in a 30 × 40 cm open field enclosure for 10 min using transmitted light and all movement was recorded with a Logitech C930e camera. For the hypoxia group, the analyses were carried out in the hypoxia workstation at 15% O_2_ (dimensions: 2337 mm L × 762 mm D, Hypoxic Glove Box, Coy Laboratory Products, USA). The recordings were analyzed with Ethovision 14 software, and the distance traversed and the time spent in the open field center were quantified. After six weeks of the treatment, the mice were sacrificed using 30 µl per g of body weight fentanyl/midazolam/medetomidine. At sacrifice, a blood sample was taken from *vena cava*, the body was perfused with ice cold 0.9% NaCl for 3 min through the left ventricle of the heart and the organs were collected.

### Immunohistochemistry

5 µm sections from the formalin-fixed paraffin-embedded tissues were cut, immunohistochemically stained with Dako REAL EnVision Detection System (K5007, Aglinet), and viewed with Zeiss Axio Imager motorized Leica DM LB2 microscope and photographed with Axiocam 506 color, Leica DCF 320 camera or Hamamatsu NanoZoomer S60 slide scanner. β-amyloid positive deposits were quantified with anti-β-amyloid antibodies (mouse and human Aβ binding ab2539, Abcam or human Aβ specific 6E10, BioLegend). The following commercially available anti-β-amyloid antibodies were also tested: H31L21 (InVitrogen), 218203 (Synaptic Systems), 17-24 (BioLegend) and MAB348 (Sigma-Aldrich). Capillaries were quantified from 10 µm paraffin-embedded sections with an anti-GLUT1 antibody (#07-140, Merck). Activated glial cells were quantified with an anti-IBA1 antibody (019-19741, Wako) and CD45 antibody (ab10558 lot. GR3422640-1, Abcam). Autophagotic cells were quantified with an anti-ATG9A antibody (ab108338, Abcam).

### Computer-aided analyses of slides

Visiopharm VIS image software was used for computer-aided analysis to quantify total GLUT1-expression area of brain and to determine the IBA1, CD45 and ATG9A co-localisation within the Aβ-positive deposit affected area (within 50 µm) and in deposit-free area in one sagittal whole brain slide.

### β-galactosidase (β-gal) staining

The *Hif-p4h-2*^*gt/gt*^ brains were cut into four ~ 2 mm sections along the coronal plate, fixed in 0.2% glutaraldehyde (GA) for 2 h and stained with 2 mg/ml 5-bromo-4-chloro-3indolyl-b-d-galactopyranoside (X-gal). The stained tissues were viewed with a Leica MZ6 microscope and photographed with Leica DFC425 camera. To detect β-gal activity histologically, the brain sections were fixed in 0.2% GA 3 × 2 h, immersed overnight in 30% sucrose frozen in cryo blocks, 25 µm cryo-cut sections were floating stained in 2 mg/ml X-gal and viewed with Zeiss Axio Imager motorized histology microscope and photographed with Axiocam 506 color camera.

### Hematoxylin–eosin staining

To analyze the adipocyte size and the amount of white adipose tissue (WAT) macrophage aggregates, 5 µm sections from formalin-fixed paraffin-embedded WAT was sectioned and stained with hematoxylin–eosin (H&E). Sections were viewed with Zeiss Axio Imager motorized histology microscope and photographed with Axiocam 506 color camera. The average size of an adipocyte and the number of macrophage aggregates were calculated using Zen blue software.

### Tissue sample preparation for biochemical and gene expression analyses

Cerebral cortex and hippocampus were snap-frozen in liquid nitrogen and homogenized in 10 ml of phosphate buffered saline (PBS) per mg tissue using TissueLyser LT (Qiagen). Homogenate was aliquoted for Aβ enzyme-linked immunosorbent assay (ELISA), Western blot and quantitative real-time PCR analyses (qPCR).

### Aβ ELISA

Protease phosphatase inhibitor cocktail (04693132001, Roche) was added to the sample homogenate aliquot and the sample was diluted 1:6 in 6 M guanidine to solubilize Aβ. The total concentration of Aβ_42_ load was determined using Human/Rat Beta Amyloid ELISA Kit (Wako, High-Sensitive, 292-64501) according to the manufacturer’s instructions. The final dilution factors were 1:2000 (4 months) and 1:60,000 (10 months) and the absorbance was measured using a Tecan infinite M1000 pro plate reader.

### qPCR analyses

Total RNA was isolated from the homogenate samples using E.Z.N.A. Total RNA Kit II (Omega Bio-Tek) and reverse transcribed with an iScript cDNA synthesis Kit (Bio-Rad). qPCR was performed with iTaqSYBR Green Supermix with ROX (Bio-Rad) with the primers shown in the Supplemental Table 3.

### Western blot analyses

Protease phosphatase inhibitor cocktail (04693132001, Roche) was added to the sample homogenate aliquot. 50 µg of protein from cerebral cortex or hippocampus were resolved by SDS-PAGE gel, blotted and probed by anti-BACE1 (1:500, #PA1-757, Invitrogen) or anti-β-actin (1:5000, NB600-501, Novus Biologicals). 20 µg of protein were resolved by SDS-PAGE gel, blotted and probed by anti-C-terminal APP (1:4000, A8717, Sigma). Protein lysates for HIF1α detection were extracted with the NER-PER-kit (Pierce) according to the manufacturer’s instructions. 20 µg nuclear extracts were resolved by SDS-PAGE gel, blotted and probed by anti-HIF1α (1:500, NB100-479, Novus Biologicals). HRP-conjugated secondary anti-rabbit or anti-mouse antibodies (1:5000, DAKO, Bio-Rad to detect anti-HIF1α) were used to detect the primary antibody. The Pierce ECL system (ThermoScientific) was used for detection. Results were semi-quantified using Fiji (ImageJ) software.

### Determination of triglycerides in serum and liver tissue

Hepatic lipids were extracted overnight in an ethanol-KOH solution at 55 °C, followed by centrifugating at 10,000 *g* for 5 min. The supernatant or serum were used to assay triglycerides by an enzymatic method (Roche diagnostics) and the absorbance was measured using a Tecan infinite M1000 pro plate reader.

### Determination of glycogen in liver tissue

Liver tissue was snap-frozen in liquid nitrogen and homogenized in 10 ml phosphate buffered saline (PBS) per mg tissue using TissueLyser LT (Qiagen). Protease phosphatase inhibitor cocktail (04693132001, Roche) was added to the sample homogenate aliquot and glycogen concentration was determined using Cayman Chemical Glycogen Assay Kit (700480, Cayman Chemicals) according to manufacturer’s instructions using a dilution factor 1:100. Luminescence was measured using a Tecan infinite M1000 pro plate reader.

### Blood and serum analyses

Blood glucose concentrations were measured with a glucometer, lactate levels with a lactometer (Lactate Scout + -meter, SensLab/EKF Diagnostics) and the hemoglobin levels with a hemoglobin meter (Triolab HemoCue Hb 201 +) at sacrifice. Insulin levels were determined from serum using an insulin kit (90080 CrystalChem) according to the manufacturer’s instructions and a Tecan infinite M1000 pro plate reader. The homeostatic model assessment for insulin resistance (HOMA-IR) values were calculated with the formula: blood glucose (mmol/l) * serum insulin (pmol/l))/156.65. Serum cholesterol, HDL cholesterol and triglyceride levels were determined using reagents 04718917, 07528604 and 46219201, respectively (Roche) according to the manufacturer’s instructions, and the absorbances were measured using a Tecan infinite M1000 pro plate reader.

### Statistical analyses

Student’s two-tailed *t* test was used for analysis of statistical differences between two groups and was indicated with label *. Two-way ANOVA was used for comparison of four groups and three-way ANOVA for comparison of eight groups. Tukey’s multiple comparison test was used in ANOVA analyses and was indicated with label #. Pearson’s correlation coefficient was used to compare linear dependences between two variables. All data are presented as mean ± SEM and *P* ≤ 0.05 was considered statistically significant.

## Supplementary Information

Below is the link to the electronic supplementary material.

Supplementary file1 (PDF 949 KB)

## Data Availability

All data are provided in the paper and supplementary information.
