# Supplementary material for: Activation of the hypoxia response protects mice from amyloid-β accumulation
Source: Cell Mol Life Sci. 2022 Jul 19;79(8):432. doi: 10.1007/s00018-022-04460-6 (PMC9296391; doi:10.1007/s00018-022-04460-6)
Supplement: Supplementary file 1 — Supplementary file1 (PDF 949 KB) [file 18_2022_4460_MOESM1_ESM.pdf]

Supplementary data

Activation of the hypoxia response protects mice from amyloid-β accumulation

Teemu Ollonen[1*], Margareta Kurkela[1*], Anna Laitakari[1], Samuli Sakko[1], Henna Koivisto[2], Johanna Myllyharju[1], Heikki Tanila[2], Raisa Serpi[1], and Peppi Koivunen[1]

[1]Biocenter Oulu, Faculty of Biochemistry and Molecular Medicine, Oulu Center for Cell-Matrix Research, University of Oulu, Oulu, Finland. [2]A.I. Virtanen Institute for Molecular Sciences, University of Eastern Finland, Kuopio, Finland.

*These authors contributed equally

Short title: Hypoxia and amyloid-β

Correspondence to: Peppi Koivunen, MD, PhD, Faculty of Biochemistry and Molecular Medicine, University of Oulu, Aapistie 7C, P.O. Box 5400, FIN-90014, Finland, Email peppi.koivunen@oulu.fi,Tel. +358-8-5375822

Supplemental Table 1. Pearson correlation coefficients of anthropometric and metabolic parameters and mRNA levels of all mice. WAT, white adipose tissue weight; liver, liver weight; b, blood; C, cortical; Aβ, amyloid-β; trigly, triglycerides; glyc, glycogen; chol, cholesterol; WAT macroph, WAT macrophage aggregates; H, hippocampal. Significant associations (P<0.05) are indicated by bold.

4 mo correlation

	WAT	Liver	b-Glucose	b-Lactate	s-Insulin	b-Hemoglobin	Body weight	HOMA-IR	C Aβ	H Aβ	Liver Trigly	Liver Glyc	s-Trigly	s-Chol	s-HDL	C Xbp1	C Hspa5	C Atf6	C Eif2ak3	H Xbp1	H Hspa5	H Atf6	H Eif2ak3	C Trem2	H Trem2	C cFOS	H cFOS	C BTG2	H BTG2	C Arc	H Arc	C Dusp1	H Dusp1
WAT																																	
Liver	**0.56**																																
b-Glucose	**0.41**	0.30																															
b-Lactate	-0.09	-0.39	-0.07																														
s-Insulin	**0.53**	**0.43**	0.09	0.10																													
b-Hemoglobin	-0.18	-0.22	-0.15	0.21	-0.04																												
Body weight	0.37	**0.56**	0.05	**-0.44**	0.22	-0.27																											
HOMA-IR	**0.62**	**0.50**	**0.46**	0.08	**0.90**	-0.10	0.20																										
C Aβ	-0.02	0.04	**-0.46**	-0.35	**0.40**	0.04	**0.42**	0.12																									
H Aβ	0.07	0.06	**-0.40**	-0.35	**0.45**	0.19	0.32	0.17	**0.63**																								
Liver Trigly	**0.52**	0.20	0.36	-0.14	0.20	-0.16	0.00	0.34	-0.25	-0.07																							
Liver Glyc	**0.42**	**0.46**	0.35	-0.07	0.33	-0.27	0.26	**0.45**	0.13	0.17	0.10																						
s-Trigly	0.08	0.07	-0.07	-0.05	0.33	-0.03	0.12	0.23	**0.48**	**0.46**	-0.05	0.23																					
s-Chol	**0.67**	**0.58**	**0.62**	-0.18	0.23	-0.12	0.26	**0.46**	**-0.48**	-0.36	**0.41**	**0.51**	0.04																				
s-HDL	**0.53**	**0.42**	**0.53**	0.00	0.21	-0.19	0.21	**0.40**	-0.24	-0.25	0.12	0.54	0.19	**0.77**																			
C Xbp1	0.32	**0.52**	0.10	0.00	0.20	-0.24	0.15	0.32	-0.05	0.11	-0.11	**0.71**	0.07	0.20	0.05																		
C Hspa5	0.31	**0.56**	0.05	-0.07	0.14	**-0.63**	**0.41**	0.18	0.13	0.04	-0.10	**0.55**	0.08	0.21	0.09	**0.81**																	
C Atf6	0.11	**0.41**	0.23	0.08	0.11	-0.21	-0.04	0.31	0.03	-0.06	-0.01	**0.58**	0.16	0.26	0.05	**0.75**	**0.73**																
C Eif2ak3	0.01	0.31	0.08	-0.10	-0.03	-0.16	-0.04	0.10	0.08	-0.07	-0.15	**0.57**	0.06	0.03	0.09	**0.71**	**0.68**	**0.77**															
H Xbp1	0.14	0.36	0.08	-0.06	0.10	-0.28	0.13	0.20	0.04	-0.10	-0.37	**0.57**	0.17	-0.01	-0.12	**0.79**	**0.73**	**0.67**	**0.59**														
H Hspa5	**0.49**	**0.54**	0.13	0.13	0.32	**-0.50**	0.33	**0.48**	0.04	-0.02	-0.17	**0.58**	-0.01	0.30	0.13	**0.76**	**0.81**	**0.59**	**0.48**	**0.65**													
H Atf6	0.01	0.14	-0.12	**-0.59**	0.04	-0.02	0.16	-0.05	**0.47**	0.23	-0.11	0.33	0.18	-0.27	-0.22	0.38	**0.46**	0.37	**0.63**	**0.55**	0.22												
H Eif2ak3	0.05	0.35	0.01	**-0.64**	-0.16	-0.24	0.24	-0.21	0.16	0.38	-0.10	0.44	0.27	0.02	-0.03	0.44	**0.46**	0.32	**0.48**	**0.48**	0.12	**0.70**											
C Trem2	0.14	0.13	-0.10	**-0.42**	0.06	-0.12	0.38	-0.01	**0.46**	0.00	-0.10	0.00	-0.16	-0.16	-0.05	0.09	0.34	0.06	**0.47**	0.14	0.12	**0.68**	0.27										
H Trem2	0.24	0.15	-0.31	**-0.49**	0.38	0.02	**0.57**	0.12	**0.76**	**0.54**	-0.07	0.14	0.39	-0.39	-0.19	-0.04	0.12	-0.14	0.00	0.15	-0.02	**0.62**	0.36	**0.53**									
C cFOS	-0.16	-0.27	0.00	-0.15	-0.16	0.06	-0.38	-0.14	0.08	-0.34	0.43	-0.32	-0.13	0.00	-0.06	-0.42	-0.17	0.02	0.17	-0.39	-0.27	0.19	-0.17	0.39	-0.09								
H cFOS	-0.20	-0.14	0.07	-0.29	-0.30	-0.24	-0.22	-0.36	-0.17	-0.15	0.52	-0.27	-0.23	-0.12	-0.13	-0.05	0.13	-0.03	0.26	-0.11	-0.10	0.30	0.22	0.34	-0.14	**0.61**							
C BTG2	-0.26	**-0.46**	-0.22	0.09	0.05	0.22	-0.40	-0.07	0.15	-0.22	0.38	-0.57	-0.09	-0.19	-0.27	-0.64	-0.37	-0.18	-0.15	**-0.50**	**-0.42**	-0.01	-0.33	0.16	-0.05	**0.88**	**0.41**						
H BTG2	-0.14	-0.23	0.00	-0.37	-0.10	0.06	-0.10	-0.20	0.12	0.01	0.51	-0.37	-0.11	-0.34	-0.20	-0.26	-0.11	-0.21	0.18	-0.26	-0.36	**0.40**	0.19	**0.55**	0.19	**0.58**	**0.84**	**0.47**					
C Arc	0.06	0.37	**0.40**	-0.15	-0.08	-0.45	-0.07	0.10	-0.10	-0.19	0.37	0.32	0.20	0.33	0.21	0.10	0.23	0.26	0.11	0.05	0.34	0.00	-0.04	-0.19	-0.26	**0.54**	**0.50**	0.22	0.11				
H Arc	-0.28	-0.30	-0.09	-0.31	-0.18	-0.01	-0.25	-0.32	-0.02	-0.21	0.37	-0.34	-0.30	-0.26	-0.27	-0.24	-0.06	-0.24	0.15	-0.14	-0.18	0.41	0.16	**0.46**	0.03	**0.68**	**0.85**	**0.58**	**0.79**	0.35			
C Dusp1	-0.16	-0.33	0.03	-0.10	-0.21	0.09	-0.50	-0.19	-0.22	-0.41	0.54	-0.34	-0.30	-0.04	-0.13	-0.26	-0.19	-0.10	0.10	-0.22	-0.30	0.18	-0.05	0.17	-0.21	**0.80**	**0.78**	**0.67**	**0.69**	**0.63**	**0.84**		
H Dusp1	-0.23	0.15	-0.03	-0.07	-0.19	-0.34	-0.08	-0.13	-0.20	-0.73	-0.08	-0.17	-0.40	-0.04	-0.11	-0.11	0.05	**0.55**	**0.48**	0.08	0.23	-0.26	**-0.61**	-0.18	**-0.51**	**0.65**	0.11	**0.56**	-0.16	**0.51**	0.22	**0.40**	

10 mo correlation

	WAT	Liver	b-Glucose	b-Lactate	s-Insulin	b-Hemoglobin	Body weight	HOMA-IR	C Aβ	H Aβ	C Bace1	Liver Trigly	Liver Glyc	s-Trigly	s-Chol	s-HDL	s-LDL	WAT macroph	C Xbp1	C Hspa5	C Atf6	C Eif2ak3	H Xbp1	H Hspa5	H Atf6	H Eif2ak3	C Trem2	H Trem2	C cFOS	H cFOS	C BTG2	H BTG2	C Arc	H Arc	C Dusp1	H Dusp1	C GLUT1
WAT																																					
Liver	**0.68**																																				
b-Glucose	**0.53**	**0.49**																																			
b-Lactate	0.18	0.07	0.33																																		
s-Insulin	**0.61**	**0.49**	**0.51**	-0.01																																	
b-Hemoglobin	-0.08	0.05	-0.10	-0.18	0.08																																
Body weight	**0.93**	**0.69**	**0.50**	0.14	**0.70**	0.01																															
HOMA-IR	**0.63**	**0.55**	**0.72**	0.12	**0.95**	0.06	**0.67**																														
C Aβ	0.12	0.00	-0.07	-0.06	0.02	-0.24	0.02	-0.02																													
H Aβ	-0.08	0.04	-0.25	-0.15	-0.22	-0.11	-0.11	-0.21	**0.63**																												
C Bace1	0.03	0.06	0.06	-0.03	0.26	0.05	0.09	0.25	-0.07	-0.11																											
Liver Trigly	**0.66**	**0.46**	**0.49**	0.15	**0.61**	0.09	**0.67**	**0.66**	0.24	-0.11	0.30																										
Liver Glyc	0.02	-0.01	0.09	0.23	0.06	**-0.47**	0.00	0.07	0.38	-0.09	-0.14	-0.13																									
s-Trigly	0.16	-0.04	0.01	0.24	0.23	0.31	0.22	0.17	-0.10	-0.19	0.25	0.28	-0.16																								
s-Chol	**0.62**	**0.52**	**0.49**	0.17	0.39	-0.09	**0.55**	**0.45**	0.36	0.01	0.02	0.37	0.17	0.02																							
s-HDL	0.18	0.33	0.15	0.26	-0.01	0.19	0.13	0.03	0.16	0.04	-0.15	0.20	-0.08	0.25	0.22																						
s-LDL	0.18	0.01	0.15	-0.16	0.22	-0.25	0.18	0.22	0.07	-0.02	0.13	0.01	0.18	-0.30	0.38	**-0.82**																					
WAT macroph	0.29	0.19	0.12	-0.02	0.28	-0.03	0.31	0.22	0.25	0.23	0.04	0.12	0.05	-0.16	0.07	-0.24	0.28																				
C Xbp1	-0.24	-0.15	-0.33	-0.21	-0.09	**-0.41**	-0.13	-0.20	0.11	-0.07	-0.07	-0.39	**0.48**	-0.47	0.00	**-0.59**	**0.68**	0.15																			
C Hspa5	-0.02	-0.03	0.22	-0.12	0.20	0.07	-0.05	0.16	-0.05	-0.05	0.11	0.18	-0.05	0.35	0.02	-0.03	0.02	0.04	0.28																		
C Atf6	-0.06	0.13	-0.10	0.04	-0.08	**-0.47**	-0.11	-0.09	0.06	-0.10	-0.36	-0.23	0.29	**-0.58**	0.19	**-0.44**	**0.66**	0.22	**0.58**	**0.58**																	
C Eif2ak3	-0.17	-0.05	-0.27	0.12	-0.24	-0.48	-0.11	-0.27	0.15	-0.15	-0.50	-0.20	0.35	-0.45	0.04	-0.42	0.52	0.24	0.61	0.45	0.72																
H Xbp1	**0.48**	**0.54**	0.17	0.08	**0.48**	-0.01	**0.62**	0.39	-0.16	-0.17	0.36	0.28	0.27	-0.23	0.35	0.02	0.23	0.28	0.32	**0.64**	0.18	0.16															
H Hspa5	0.37	0.26	0.17	0.19	0.28	-0.35	0.37	0.24	-0.15	-0.33	0.39	0.16	**0.46**	-0.15	0.36	-0.01	0.26	0.19	**0.41**	**0.65**	0.21	0.28	**0.79**														
H Atf6	**0.53**	**0.41**	**0.51**	0.29	**0.68**	0.06	**0.51**	**0.67**	-0.36	-0.36	**0.45**	0.34	0.14	0.22	0.35	0.19	0.01	0.24	0.01	0.27	-0.05	-0.24	**0.67**	**0.65**													
H Eif2ak3	**0.44**	**0.45**	0.22	0.16	0.35	-0.04	**0.49**	0.33	**-0.51**	-0.34	0.32	0.10	0.13	-0.16	0.14	-0.04	0.15	0.20	0.22	**0.53**	0.15	0.09	**0.81**	**0.78**	**0.79**												
C Trem2	0.25	0.15	0.17	-0.03	0.37	**-0.41**	0.19	0.31	**0.41**	-0.07	0.04	0.29	**0.74**	-0.10	**0.46**	0.05	0.26	0.16	**0.46**	0.37	0.34	0.30	**0.46**	**0.54**	0.32	0.22											
H Trem2	-0.04	0.19	-0.24	-0.17	-0.26	-0.33	-0.07	-0.26	0.30	**0.76**	0.27	-0.32	-0.08	-0.17	0.05	-0.15	0.21	0.31	0.20	0.15	0.11	-0.07	0.21	0.05	0.05	0.07	0.08										
C cFOS	-0.09	-0.27	0.08	-0.14	-0.15	0.35	-0.22	-0.05	0.08	0.03	-0.35	0.18	**-0.49**	0.36	-0.20	0.25	**-0.44**	-0.34	-0.48	**-0.45**	**-0.41**	-0.22	**-0.66**	**-0.58**	**-0.49**	**-0.67**	-0.33	-0.35									
H cFOS	-0.03	-0.22	0.03	-0.04	-0.06	**0.40**	-0.12	0.01	**-0.48**	-0.33	0.03	0.06	**-0.54**	0.39	-0.32	0.07	-0.32	**-0.42**	-0.28	-0.15	-0.38	-0.34	-0.15	-0.02	0.03	0.01	-0.38	**-0.40**	**0.60**								
C BTG2	-0.09	-0.27	-0.04	-0.03	-0.19	0.25	-0.27	-0.11	0.09	0.07	-0.26	0.10	**-0.57**	0.33	-0.17	0.23	**-0.40**	-0.28	-0.44	**-0.41**	-0.35	-0.24	**-0.56**	**-0.44**	**-0.40**	**-0.56**	-0.36	-0.25	**0.91**	**0.70**							
H BTG2	0.21	0.09	0.15	0.13	0.02	0.26	0.10	0.11	**-0.57**	**-0.46**	0.08	0.18	-0.35	0.14	0.03	0.13	-0.14	-0.18	-0.15	0.10	-0.17	-0.05	0.33	**0.47**	**0.41**	**0.55**	-0.08	-0.39	0.15	**0.69**	0.24						
C Arc	-0.14	-0.30	0.05	-0.07	-0.19	0.17	-0.31	-0.09	0.12	0.07	-0.35	0.10	-0.45	0.37	-0.20	0.24	-0.43	-0.36	-0.48	**-0.45**	-0.31	-0.22	**-0.72**	**-0.59**	**-0.51**	**-0.71**	-0.30	-0.27	**0.97**	**0.55**	**0.92**	0.04					
H Arc	0.12	-0.08	0.22	0.13	0.06	0.37	0.04	0.17	**-0.44**	-0.33	0.00	0.24	-0.60	0.38	-0.09	0.00	-0.09	-0.31	-0.24	0.02	-0.29	-0.12	0.00	0.12	0.15	0.13	-0.35	-0.32	**0.51**	**0.86**	**0.59**	**0.73**	**0.44**				
C Dusp1	-0.34	-0.31	0.07	-0.29	-0.28	0.04	-0.37	-0.19	0.08	0.21	-0.18	-0.16	-0.29	0.26	-0.21	0.04	-0.22	-0.23	-0.27	**-0.53**	-0.30	-0.23	**-0.83**	**-0.70**	**-0.55**	**-0.79**	-0.32	0.04	**0.78**	0.09	**0.73**	-0.34	**0.85**	0.02			
H Dusp1	0.13	0.02	0.18	0.05	-0.08	0.16	0.00	0.05	**-0.44**	-0.27	0.11	0.07	**-0.51**	0.21	0.03	0.02	-0.02	-0.31	-0.06	0.19	-0.13	-0.08	0.10	0.31	0.20	0.23	-0.23	-0.12	0.37	**0.81**	**0.47**	**0.74**	0.33	**0.89**	0.01		
C GLUT1	-0.21	-0.30	-0.11	-0.35	-0.18	0.30	-0.13	-0.15	**-0.51**	-0.36	0.04	-0.02	**-0.42**	0.24	**-0.81**	-0.21	-0.39	0.05	**-0.48**	-0.31	-0.34	-0.18	-0.34	-0.38	-0.23	-0.12	**-0.63**	-0.38	**0.48**	**0.61**	**0.51**	0.21	**0.44**	0.34	0.28	0.13	

Supplemental Table 2 RNA sequencing (RNASeq) analysis of the cerebral cortex of the 4-month-old hypoxia and normoxia treated APP/PSEN1 mice (n = 2 + 2). Genes with lowest adjusted *P* values for a difference in expression are indicated. The Log2FoldChange displays the difference in expression level between hypoxia mice and normoxia. The adjusted *P* value displays the significance of the difference in expression levels.

Gene	Log2FoldChange	*P* value adjusted
Mfrp	8.4104	0.0054
Dusp1	1.0289	0.0110
Shox2	4.8264	0.0112
Btg2	1.1773	0.1052
c-Fos	1.0255	0.1119
Eif2s3y	-1.0067	0.2517
Ciart	1.2509	0.2517
Arc	0.6472	0.2517

Supplemental Table 3 Primers used in the quantitative PCR analyses.

Gene	Forward primer (5' -> 3')	Reverse primer (5' -> 3')
Arc	AAGTGCCGAGCTGAGATGC	CGACCTGTGCAACCCTTTC
Atf6	TCGCCTTTTAGTCCGGTTCTT	GGCTCCATAGGTCTGACTCC
Bace1	GGAACCCATCTCGGCATCC	TCCGATTCCTCGTCGGTCTC
Btg2	ATGAGCCACGGGAAGAGAAC	GCCCTACTGAAAACCTTGAGTC
Dusp1	GTTGTTGGATTGTCGCTCCTT	TTGGGCACGATATGCTCCAG
Eif2ak3	AGTCCCTGCTCGAATCTTCCT	TCCCAAGGCAGAACAGATATACC
c-Fos	CGGGTTTCAACGCCGACTA	TTGGCACTAGAGACGGACAGA
Hif-p4h-2	GCGTCCCAGTCTTTATTTAGATA	CTGGGCAACTACAGGATAAAC
Hspa5	ACTTGGGGACCACCTATTCT	ATCGCCAATCAGACGCTCC
Trem2	TGGAACCGTCACCATCACTC	TGGTCATCTAGAGGGTCCTCC
Xbp1	AGCTTTTACGGGAGAAAACTCAC	CCTCTGGAACCTCGTCAGGA

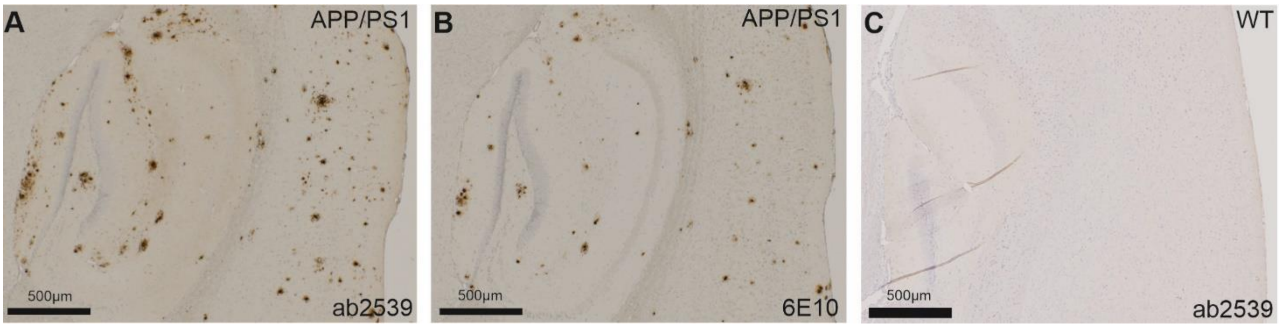

Fig. S1. Validation of the Aβ antibody. (**A**) Mouse/human Aβ binding antibody ab2539 detects amyloid plaques consisting of mixed mouse and human Aβ correspondingly to (**B**) human Aβ specific 6E10 in the cortex and hippocampus of the APP/PSEN1 mice but not in wild type (WT) (**C**).

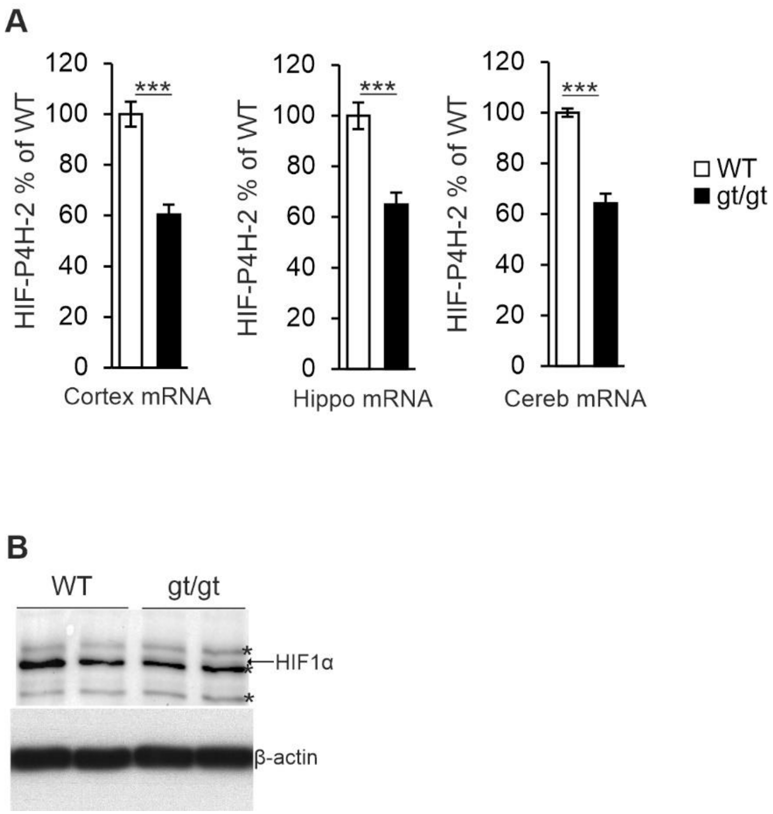

Fig. S2. HIF1α is stabilized in *Hif-p4h-2*$^{gt/gt}$ brain. HIF stabilisation in wild-type (WT) and *Hif-p4h-2*$^{gt/gt}$ (gt/gt) brain. (**A**) qPCR analysis of *Hif-p4h-2* mRNA in cortex, hippocampus and cerebellum of WT and gt/gt mice. Data are means ± SEM. ***P < 0.005 in T-test. n = 10 WT, 10 gt/gt. (**B**) Western blot analysis of HIF1α in hippocampus of WT and gt/gt mice. *indicates non-specific bands. β-actin was used as a loading control.

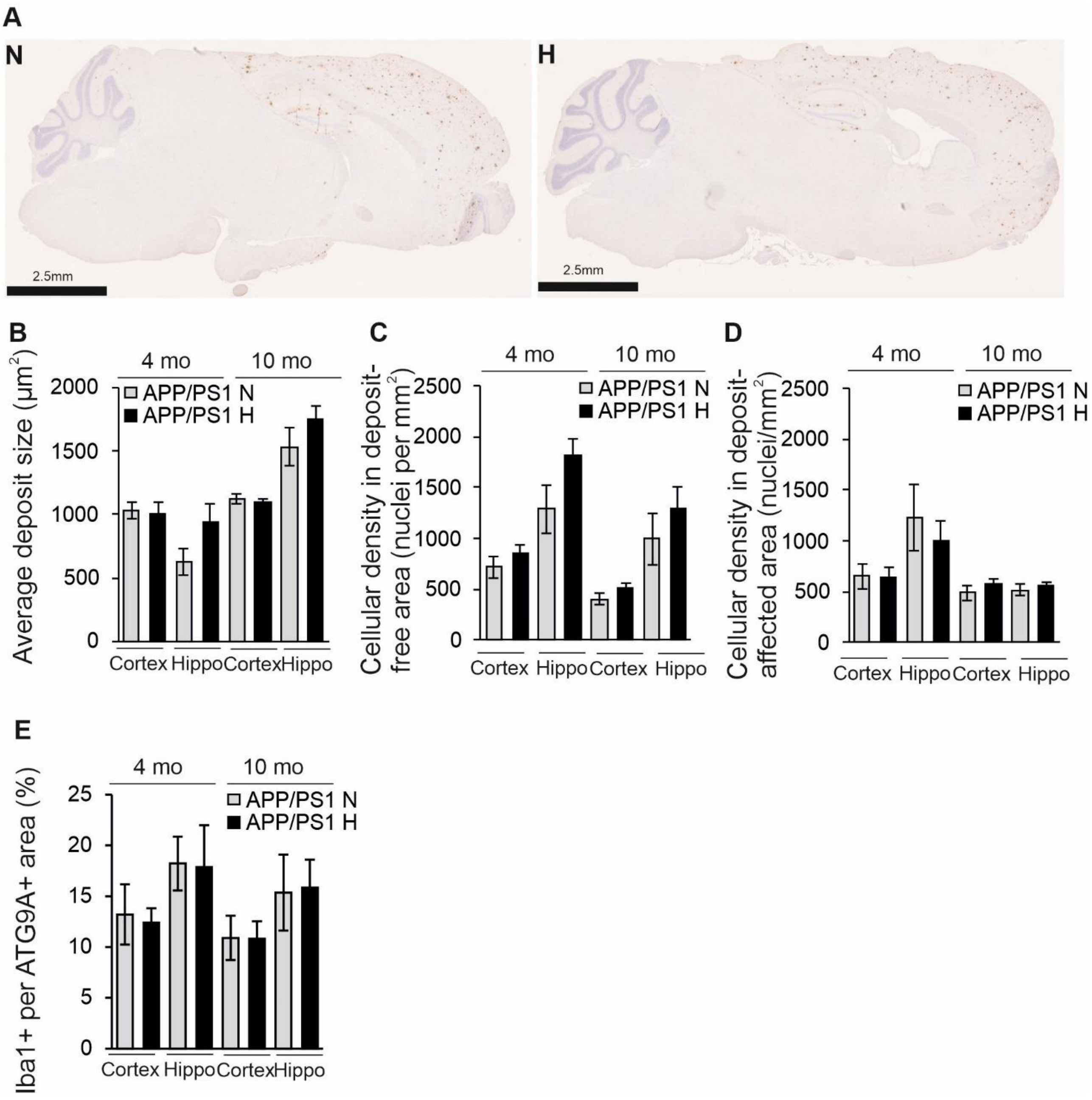

Fig. S3. Immunohistochemical analysis of Aβ plaques, nuclear density, dystrophic neurites and microglia in normoxia and hypoxia treated APP/PSEN1 cortex and hippocampus. (**A**) Examples of immunohistochemical stainings of sagittal brain sections of 10-month-old APP/PS1 mice with the human specific 6E10 antibody. (**B**) Quantification of average size of Aβ positive plaques. Total cellular density in deposit-free area (**C**) and deposit-affected area (**D**). IBA1-positive area in ATG9A-positive area (**E**). n = 8 APP/PSEN1 N 4 mo, n = 9-10 APP/PSEN1 H 4 mo, n = 8-9 APP/PSEN1 N

10 mo, n = 9-11 APP/PSEN1 H 10 mo. Abbreviations: Hippo, hippocampus; H, hypoxia; mo, months old; N, normoxia.

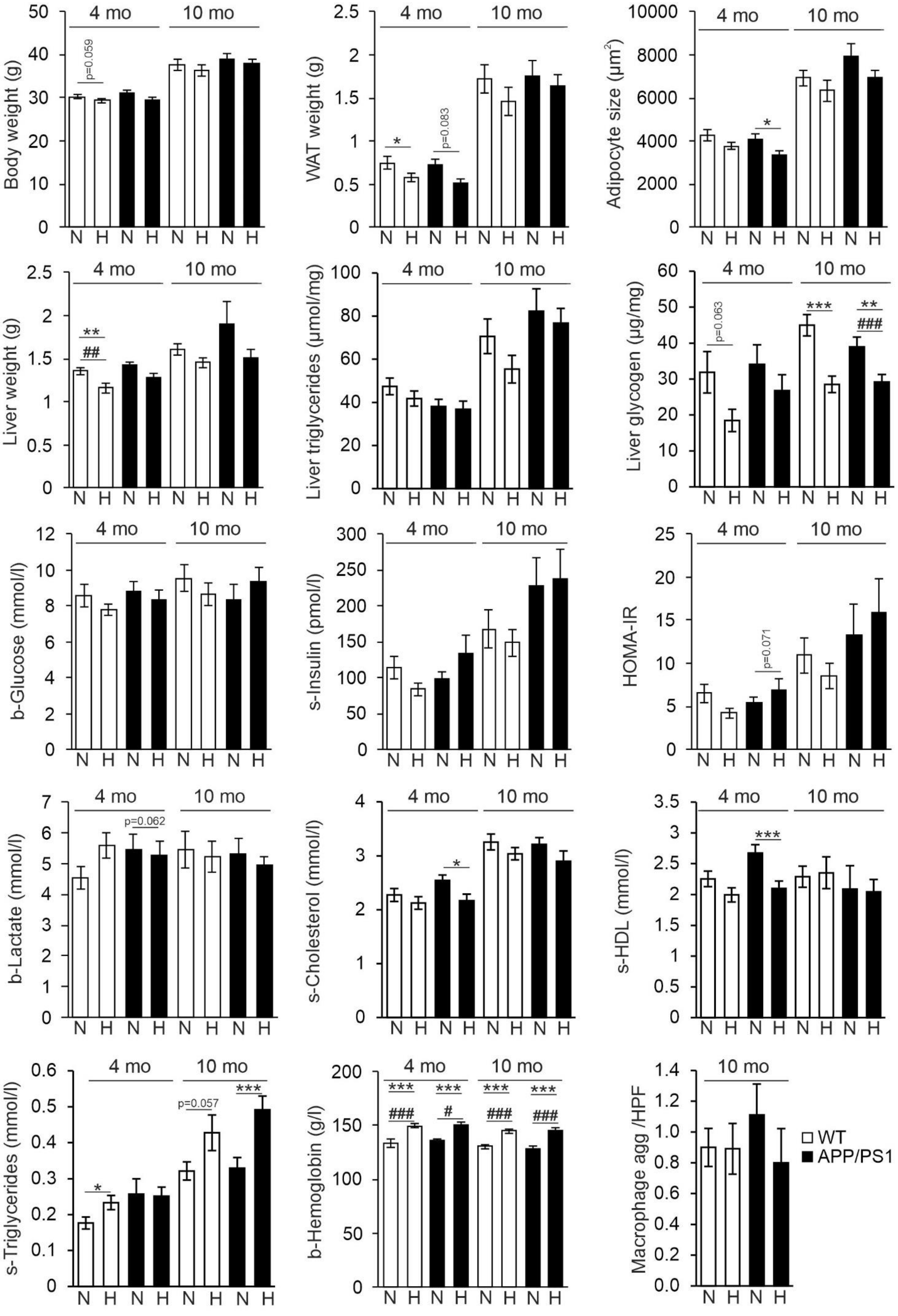
4 mo
10 mo
Body weight (g)
p=0.059
WAT weight (g)
*
p=0.083
Adipocyte size (µm2)
*
Liver weight (g)
**
##
Liver triglycerides (µmol/mg)
Liver glycogen (µg/mg)
p=0.063
***
**
###
b-Glucose (mmol/l)
s-Insulin (pmol/l)
HOMA-IR
p=0.071
b-Lactate (mmol/l)
p=0.062
s-Cholesterol (mmol/l)
*
s-HDL (mmol/l)
***
s-Triglycerides (mmol/l)
*
p=0.057
***
b-Hemoglobin (g/l)
***
###
#
Macrophage agg /HPF
N H N H N H N H
WT
APP/PS1

Fig. S4. Hypoxia treatment improves metabolic parameters in APP/PSEN1 mice. Metabolic parameters of wild-type (WT) and APP/PSEN1 mice after 6 weeks of hypoxia treatment. Data are means ± SEM. **P* < 0.05, ***P* < 0.01, ****P* < 0.005 in T-test, #*P* < 0.05, ##*P* < 0.01, ###*P* < 0.005 in 2-way ANOVA. n = 14 WT N 4 mo, 12 WT H 4 mo, 8 APP/PSEN1 N 4 mo, 9-10 APP/PSEN1 H 4 mo, 13-14 WT N 10 mo, 10-11 WT H 10 mo, 9 APP/PSEN1 N 10 mo, 10-11 APP/PSEN1 H 10 mo. Abbreviations: agg, aggregates; b, blood; HPF, high power field; H, hypoxia; mo, months old; N, normoxia; s, serum; WAT, white adipose tissue.

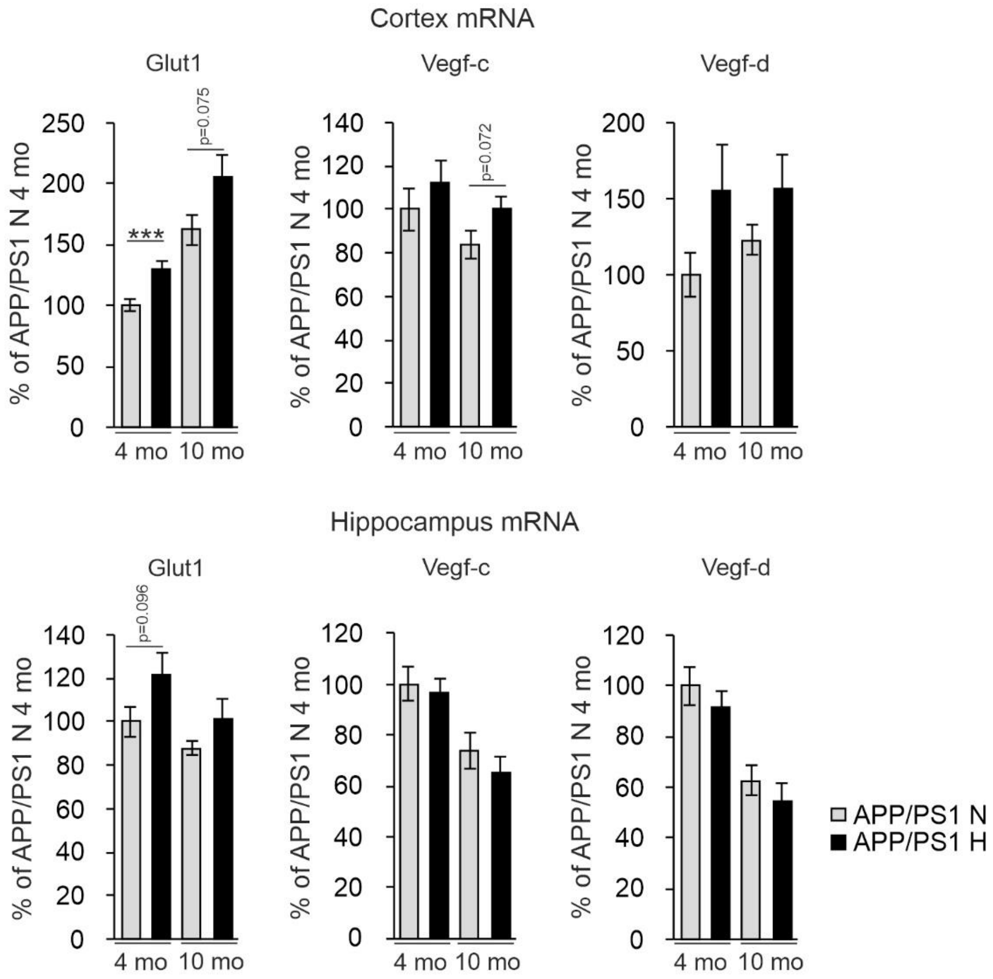

Fig. S5. Expression of angiogenetic mRNAs in the APP/PSEN1 brain following hypoxic exposure. mRNA expression after 6 weeks of normoxia or hypoxia treatment in cortex and hippocampus of APP/PSEN1 mice. Data are means ± SEM. ****P*<0.005 in T-test. n = 8 N 4 mo, 9-10 H 4 mo, 8-9 N 10 mo, 10-11 H 10 mo. Abbreviations: H, hypoxia; mo, months old; N, normoxia.
